# Parsimonious model for mass-univariate vertexwise analysis

**DOI:** 10.1117/1.JMI.9.5.052404

**Published:** 2022-05-20

**Authors:** Baptiste Couvy-Duchesne, Futao Zhang, Kathryn E. Kemper, Julia Sidorenko, Naomi R. Wray, Peter M. Visscher, Olivier Colliot, Jian Yang

**Affiliations:** a University of Queensland, Institute for Molecular Bioscience, St. Lucia, Queensland, Australia; b Sorbonne University, Paris Brain Institute (ICM), CNRS, INRIA, INSERM, AP-HP, Hôpital de la Pitié Salpêtrière, Paris, France; c Westlake University, School of Life Sciences, Hangzhou, China; d Westlake Laboratory of Life Sciences and Biomedicine, Hangzhou, China

**Keywords:** structural brain MRI, vertex-wise processing, linear mixed model, association, brain mapping

## Abstract

**Purpose:**

Covariance between gray-matter measurements can reflect structural or functional brain networks though it has also been shown to be influenced by confounding factors (e.g., age, head size, and scanner), which could lead to lower mapping precision (increased size of associated clusters) and create distal false positives associations in mass-univariate vertexwise analyses.

**Approach:**

We evaluated this concern by performing state-of-the-art mass-univariate analyses (general linear model, GLM) on traits simulated from real vertex-wise gray matter data (including cortical and subcortical thickness and surface area). We contrasted the results with those from linear mixed models (LMMs), which have been shown to overcome similar issues in omics association studies.

**Results:**

We showed that when performed on a large sample (N=8662, UK Biobank), GLMs yielded greatly inflated false positive rate (cluster false discovery rate >0.6). We showed that LMMs resulted in more parsimonious results: smaller clusters and reduced false positive rate but at a cost of increased computation. Next, we performed mass-univariate association analyses on five real UKB traits (age, sex, BMI, fluid intelligence, and smoking status) and LMM yielded fewer and more localized associations. We identified 19 significant clusters displaying small associations with age, sex, and BMI, which suggest a complex architecture of at least dozens of associated areas with those phenotypes.

**Conclusions:**

The published literature could contain a large proportion of redundant (possibly confounded) associations that are largely prevented using LMMs. The parsimony of LMMs results from controlling for the joint effect of all vertices, which prevents local and distal redundant associations from reaching significance.

## 1 Introduction

**Fig. 1:**
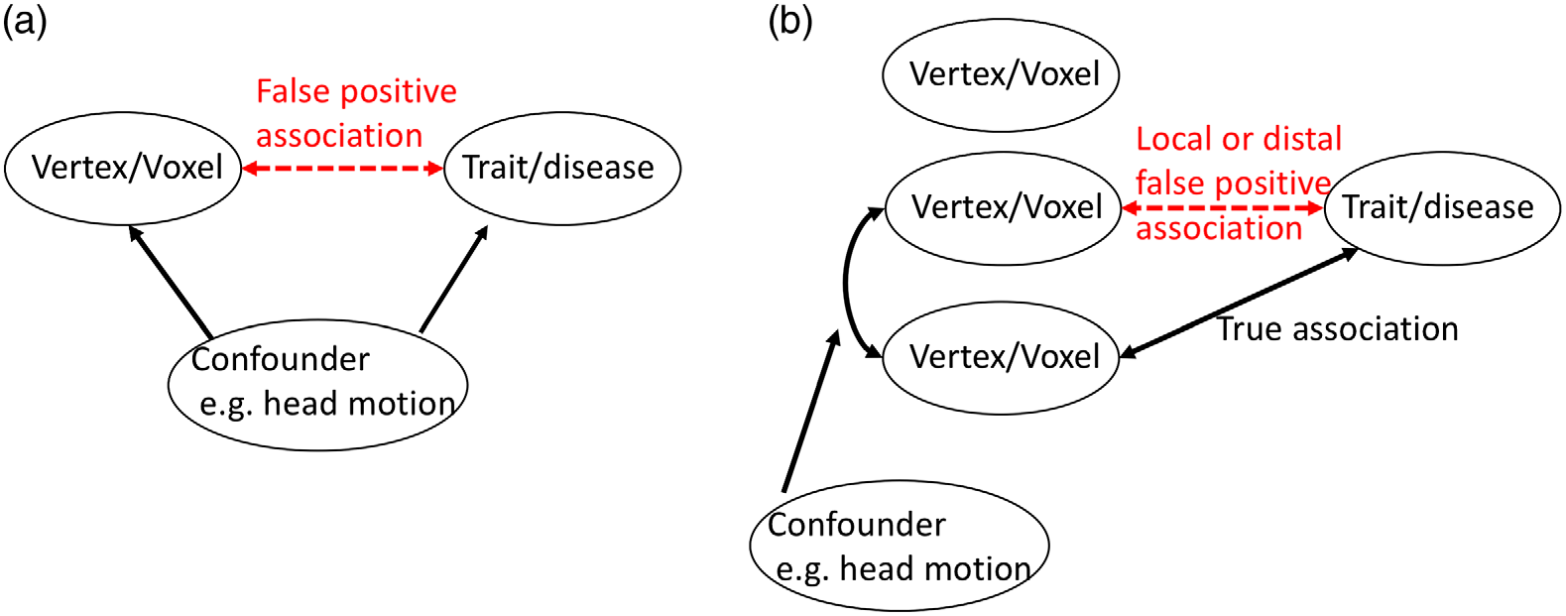
(a) Illustration of the traditional confounding paradigm (and (b) of the confounding that may arise in association studies performed across correlated brain features. One-sided arrows represent a causal effect, and two-sided arrows a correlation.

Brain MRI scans can generate hundreds of thousands of vertex/voxelwise measurements per individual, which can be linked to other measured traits/diseases using mass univariate vertex/voxelwise association analyses. Results of association analyses (and subsequent follow-up analyses) can shed light on the brain networks or cell composition relevant for the trait/disease and may be leveraged for brain-feature-based phenotype prediction. However, brain measurements may exhibit a pattern of correlation, owing to factors (e.g., head size, MRI scanner/artifact,^1^ or demographics^2^) that can generate confounded brain-trait associations. Induced local correlations with a true brain biomarker can generate a smear of association (i.e., a cluster of associated vertices), which may limit the precise localization of the directly associated regions. On the other hand, long-range vertex correlations caused or inflated by factors irrelevant to the trait of interest, may be more prejudicial, as they can yield distal false positives (Fig. 1).

Two approaches can be used to limit the inflation of false positives described above. One is to control for the confounders in the association testing, although it requires knowledge and measurement of the factors influencing (or more generally associated with) the covariance between brain measurements. Note that these factors can overlap with traditional confounders of neuroimaging studies (e.g., head size, age, sex, and head motion), and additional confounders are being identified as sample sizes increase.^3^ Another correction strategy is to control for the other vertices in the association testing, to remove the signal that could be attributed to another brain vertex or region. The difficulty of such approach is that typically, the number of vertex/voxelwise measurements (p) far exceeds the number of participants (N) in the study. The p≫N paradigm implies that the marginal joint associations with all p vertices cannot be estimated in a single general linear model (GLM).

Statistically, the challenge of mass univariate vertexwise analyses resembles that of genome-wide association studies (GWAS) or methylation-wide associations studies (MWAS), which aim to identify genomic regions associated with a phenotype in the presence of correlated features (i.e., genetic variants or DNA methylation probes). Several studies have demonstrated that feature correlation [i.e., linkage disequilibrium (LD) or population structure in genetics] can result in inflated false positive rate (FPR),^4^^–^^6^ even more so when the sample size increases.^5^ This led GLMs to be replaced by linear mixed models (LMMs)^6^^–^^8^ that co-vary out all features by fitting them as random effects. LMMs have been shown to better control the inflation of false positive associations arising from LD or correlation between probes and to minimize the occurrence of false positives in both GWAS and MWAS.^6^^,^^7^^,^^9^

LMMs are commonly used in neuroimaging to model longitudinal data.^10^ Instead, we rely here on a formulation that allows fitting the high-dimensional brain image as a single random-effect. Such LMMs allow estimation of the overall degree of association between a trait and a high-dimensional brain image, coined “morphometricity” in the context of structural brain measurements.^11^^,^^12^ Recently, we have shown that a single LMM framework was suited to estimate morphometricity in large datasets, to draw links between traits through their associations with similar brain structure (gray-matter correlation), and to build brain-based predictors.^12^ The LMMs we propose here complement our previous work by identifying the vertices/voxels that contribute to the morphometricity and phenotype prediction.

Here, we sought to evaluate whether the inflation of false positives observed in omics data is also present in neuroimaging data. In the first part of the analysis, we performed extensive simulations of continuous phenotypes from real gray-matter data to quantify FPR as well as statistical power, mapping precision and prediction accuracy achieved from mass-univariate analyses. We compared the performances of the current state-of-the-art GLMs to that of LMMs inspired by omics association studies. In the second part, we sought to characterize the brain regions associated with real phenotypes (i.e., age, sex, BMI, fluid IQ, and smoking status) that previously exhibited significant morphometricity,^12^ to confirm the results obtained on simulated traits. Our analyses relied on 14,451 MRI images collected by the UK Biobank (UKB), one of the largest brain imaging initiative.^13^

### 1.1 Novelties and Contribution

The novelties and contributions of our paper are as follows:

• We propose LMMs for brain mapping, inspired from those using in genetics, which aims at overcoming false positive issues found in standard analyses.
• By controlling for all brain measurements (fitted as a random effect), the LMMs remove redundant associations leading to more parsimonious results.
• We demonstrate that, compared with the current state-of-the-art, the LMMs minimize FPR while also maximizing power, mapping precision, and prediction accuracy.

## 2 Material and Methods

### 2.1 Models of Mass-Univariate Vertexwise Analyses

First, we considered five GLMs that differ in term of covariates used when estimating the association ($b_{i}$) between the trait and the i’th (standardized) vertexwise measurement $\left(X_{i}\right)$. They can be written under the form:

$$y=\mathrm{Zc}+X_{i}b_{i}+\varepsilon ,$$ (1)

where y is the vector of phenotype for the N individuals, Z is a matrix of size N×q of q covariates, and c is a vector of the q fixed effects.

The five GLMs are differentiated as follows: (1) GLM with no covariates (no covariates), (2) GLM including the most commonly used covariates in similar analyses: age, sex, and intracranial volume (ICV) (age, sex, ICV corrected), (3) and (4) GLMs including 5 and 10 principal components (PCs) of gray-matter variation, respectively (5 global PCs, 10 global PCs), (5) GLM including 10 PCs specific to the measurement type (cortical thickness, cortical surface, subcortical thickness, or subcortical surface area), referred to as “10 modality specific PCs.” Gray-matter PCs capture the main axes of covariations between vertices, and we expect that by controlling for them we may be able to remove unmeasured or unknown factors contributing to long-range correlation between vertices (which might include demographics, MRI machine, head motion, software update, processing option, etc.). Note that PCs from genetic data are commonly used in GWAS to limit the FPR of GLMs analyses^14^ but are rarely used in neuroimaging analyses. The difficulties of PC correction are to determine the optimal number of PCs, which controls for confounding effects without removing signals of interest. In practice, this may prove extremely difficult considering that the optimal number of PCs could depend on the trait/variable of interest, and that PCs are notoriously hard to interpret and have not been comprehensively investigated on these data. Thus, we arbitrarily chose two scenarios with the first 5 or 10 PCs. In addition, GLMs without covariates are also very rare, but worth considering to appreciate the effect of including covariates.

Finally, we considered three LMMs that can be seen as extensions of the previous approaches in that they further control for all vertexwise measurements. The first LMM model (LMM global BRM), analogous to the MOA (MLM-based omic association) model,^6^ can be written as

$$y=X_{i}b_{i}+X\beta +\varepsilon .$$ (2)

Here, X is the N×p matrix of all standardized vertexwise measurements, β is the p×1 vector of joint vertex-trait associations. β is a vector of random effects, allowing for p>N, with $\beta ∼N(0,I\sigma_{\beta }^{2})$, and ε is the error term assumed to follow $\varepsilon ∼N(0,I\sigma_{\varepsilon }^{2})$. $\sigma_{\beta }^{2}$ and $\sigma_{\varepsilon }^{2}$ are the variances of the random effects β and ε. The variance-covariance matrix for y is $\mathrm{var}(y)=V={\mathrm{XX}}^{\prime }\sigma_{\beta }^{2}+I\sigma_{\varepsilon }^{2}=Bp\sigma_{\beta }^{2}+I\sigma_{\varepsilon }^{2}$. Here, we regard $B=XX^{\prime }/p$ as the brain relatedness matrix and $p\sigma_{\beta }^{2}$ the morphometricity (proportion of phenotypic variance captured by all vertices).^15^

We considered a second LMM (“LMM with covariates”) that includes known covariates (age, sex and ICV) fitted as fixed effects. Thus, we can separate the effect of the random effects from that of the known covariates on the results. The model becomes:

$$y=\mathrm{Zc}+X_{i}b_{i}+X\beta +\varepsilon .$$ (3)

Our third LMM (“LMM multi. BRM”) includes 4 random effects $\left(\beta_{1},\beta_{2},\beta_{3},\beta_{4}\right)$, each corresponding to a type of vertices (cortical thickness, cortical surface area, subcortical thickness and subcortical surface area).

$$y=X_{i}b_{i}+X_{1}\beta_{1}+X_{2}\beta_{2}+X_{3}\beta_{3}+X_{4}\beta_{4}+\varepsilon .$$ (4)

This more general LMM allows the distribution of effect sizes to differ based on vertex type, rather than enforcing a single distribution over all types of measurements.^15^ Note that each random effect takes up a single degree of freedom meaning that LMMs and GLMs have a comparable (large) numbers of degrees of freedom given the same sample size.

### 2.2 Statistical Testing and Multiple Comparison

We performed a $\chi^{2}$ test of the association between a vertex ($X_{i}$) and the phenotype using that, for large sample size N, ${\left(\frac{b_{i}}{SE(b_{i})}\right)}^{2}∼\chi_{1}^{2}$ under the null hypothesis of no association. In each model (GLM or LMM), we accounted for multiple testing over the vertices using Bonferroni correction, thus setting a brain-wide significance threshold of 0.05/652,283=7.6e−8. We chose the straightforward Bonferroni correction over random field theory (RFT)^16^ as RFT requires stationarity and a smooth mesh of vertex-wise residuals, which is unlikely to be the case here (we did not apply kernel smoothing on the data as it reduced the estimated morphometricity of the UKB phenotypes^15^). In addition, RFT is not currently implemented to be performed using residuals of LMMs or across several surfaces and type of measurements. Bonferroni correction is expected to be conservative under the null hypothesis (no association) because the correlations between vertices means that the effective number is tests lower than the number of tests conducted and used for the Bonferroni correction.

### 2.3 MRI Image Processing

MRI images were mostly collected in Cheadle (for 96% of the sample) and Newcastle using a 3T Siemens Skyra machine (software platform VD13) and a 32-channel head coil^13^ (see Supplementary 1 in the Supplemental Material, for MRI sequence details).

We processed the T1w and T2 FLAIR images together to enhance the tissue segmentation in FreeSurfer 6.0,^17^ which should result in a more precise skull stripping and pial surfaces definition. When the T2 FLAIR was not acquired or not usable, we processed the T1w image alone, though a recent report showed this results in systematic differences in cortical thickness.^18^ This may represent a source of noise in the data, albeit it was limited in term of number of individuals (see quality control, Supplementary 1 in the Supplemental Material 1). We extracted vertex-wise data mapping cortical surface area and thickness (“recon-all” processing in FreeSurfer) and used the maximal resolution allowed by the software (fsaverage atlas - unsmoothed). In short, FreeSurfer segments the gray/white and gray/cerebrospinal fluid borders, which delimits the gray-matter. Surfaces are mapped onto a spherical atlas to align the cortical folding patterns of the individuals, and a tessellation is applied. Cortical thickness is calculated as the closest distance from the two gray-matter boundaries, for each vertex on the tessellated surface.^19^ Surface area is measured as the mean area of all faces that meet at a particular vertex, on the gray/white matter surface.^20^ We previously showed that this cortical processing maximized the morphometricity for a wide range of phenotypes.^15^ In other words, this cortical processing maximized the information retained by the processed MRI images. In addition, we applied the ENIGMA-shape processing,^21^^,^^22^ where subcortical structures segmented in FreeSurfer are projected onto spherical atlases to quantify vertex-wise radial thickness and log Jacobian determinant,^21^^,^^22^ which is analogous to a surface area.^23^ This yielded a vertex-wise characterization of the hippocampus, putamen, amygdala, thalamus, caudate, pallidum, and accumbens. Overall, the imaging data used in the analyses comprised 652,283 vertex measurements per individual: 299,009 for cortical thickness, another 299,034 for cortical surface area, 27,120 for subcortical thickness and 27,120 for subcortical surface area.

In a post-hoc analysis, we also utilized smoothed cortical data (surface based kernel with FWHM = 20 mm), in order to evaluate the robustness of our results to variation in the MRI processing.

### 2.4 Main Sample for Simulation and Discovery

Our final sample comprised 9890 adults with complete cortical and subcortical data, aged 62.5 on average (SD=7.5, range 44.6 to 79.6) with slightly more (52.4%) female participants (see Supplementary 1 in the Supplemental Material for participant inclusion and exclusion). Of note, 341 participants did not have an exploitable T2 image.

We performed a stringent quality control (QC) to exclude one of each pair of individuals whose brains were too similar or dissimilar relative to most other individuals, resulting in 1228 exclusions (12.4% of the sample). The main reason for this exclusion was to prevent bias in the LMM estimates, although it should also remove individuals flagged as outliers by other QC criteria (e.g. 80.6% of the participants processed using T1w only, spike-like cortical parcellation in FreeSurfer)^12^ (see Supplementary 1 in the Supplemental Material for more details on QC). Importantly, all analyses were performed on the same list of individuals (post QC) to ensure that performance of the models would be comparable.

### 2.5 Independent Samples for Prediction and Replication

Our first independent sample included an additional 4942 participants of the UKB with a T1w image (downloaded in May 2018, most participants also had an exploitable T2w). The final sample (N=4160 after processing and QC) was on average 63.1 years old (SD=7.46, range 46.1 to 80.3) with 52.1% of females.

In addition, we used the OASIS3 (Open Access Series of Imaging Studies) sample^24^ to evaluate the generalizability of the prediction. The OASIS3 dataset gathers several longitudinal MRI studies conducted in the Washington University Knight Alzheimer Disease Research Center over the past 15 years. Our final sample included 1006 unique participants after processing based on T1w images and QC. When several visits were available for a participant, we selected the one with the most phenotypic information. Participants were 71.1 years old on average (SD=9.18, range 42.6 to 95.7) and mostly female (55.5%). Almost a quarter of the participants (23.6%) had a diagnosis of Alzheimer’s disease at the time of imaging.

### 2.6 Mass-Univariate Analyses on Simulated Phenotypes

#### 2.6.1 Simulation of phenotypic traits from real gray-matter data

We simulated phenotypic traits from the UKB processed (standardized) gray-matter data, instead of relying on synthetic/simulated images. This approach ensures the vertexwise data retains a realistic correlation structure. In addition, our framework includes simulation of the phenotype under not only the null hypothesis (H0) that no vertex is associated with the phenotype but also the alternative hypothesis (H1) that a set of vertices are truly associated with the phenotype.

First, we randomly selected a set of associated vertices and drew their relative effects from a normal distribution. We then calculated the simulated phenotypes as a linear combination of the individuals’ vertex values and noise.^6^ We considered three scenarios that differ in terms of number of associated vertices and total association with the phenotype. This global association between gray-matter measurements and a trait has been coined morphometricity^11^^,^^15^ and may be expressed as the proportion of the trait variance ($R^{2}$) captured by the vertexwise measurement. Our scenarios were: (i) 10 associated vertices accounting for a phenotype morphometricity of $R^{2}=0.20$ (i.e., 20% of the trait variance); (ii) 100 associated vertices with $R^{2}=0.50$; (iii) 1000 vertices with $R^{2}=40\%$. For each scenario, we simulated 100 phenotypes.

In follow-up analyses, we simulated phenotypes using the same parameters, this time restricting the associated vertices to a single type of measurement. This allowed evaluation of the specificity of each type of measurement, which possess a unique correlation pattern. In addition, this ensures our phenotypes were not associated with cortical vertices only, which represent 90% of the vertexwise measurements.

To evaluate the effect of smoothing on our results, we simulated phenotypes from smoothed brain maps. For the ease of computation, we restricted the analysis of smoothed data to the case of 10 associated vertices ($R^{2}=0.2$). We kept the same associated vertices (and weights) as in the previous simulation from unsmoothed data. Finally, we randomly simulated 100 “null” traits, to evaluate the calibration of the models under the null hypothesis of no association. All simulations were generated using the OSCA software.^6^

#### 2.6.2 Inflation of test statistics

First, we compared the empirical distribution of $\chi^{2}$ statistics to the expected distribution, which is assumed to follow a $\chi^{2}(1)$ for non-associated (null) vertices. We considered the ratio of empirical over expected median $\chi^{2}$, known as the inflation factor (λ), which is expected to be equal to one across nonassociated vertices. We also used the nominal FPR defined as the proportion of null vertices with p-values<0.05 (expected to be 0.05). Correlation between associated and null vertices (e.g., due to confounding factors) typically result in an inflation of test statistics, which may cause null vertices to reach significance in mass-univariate analyses.

#### 2.6.3 Discoverability and mapping precision

First, we quantified the model discoverability using the true positive rate (TPR) defined as the proportion or truly associated vertices reaching significance (after Bonferroni correction). Importantly, the TPR is dependent on the FPR, which can limit comparison across models (see Sec. 3.2.4). In addition, we quantified the mapping precision of mass-univariate analyses by reporting the median size of the true positive (TP) clusters. We defined TP clusters as sets of significant contiguous vertices of the mesh that contain a TP vertex.

#### 2.6.4 False positives and statistical power

We reported the familywise error rate (FWER) defined as the proportion of replicates with at least one false positive vertex (null vertex significant after Bonferroni correction). In the presence of strong correlation between neighboring vertices, it is statistically difficult to separate a TP vertex from the flanking ones, thus we can expect an FWER greater than 5%. Hence, we also reported the cluster FWER defined as the proportion of replicates with at least one false positive cluster. FWER is more stringent than false discovery rate (FDR), implying that any false positives that remain after FWER correction would also be observed using FDR.

To account for the models’ differences in FWER, we further reported the statistical power, defined as the TPR for a set risk alpha. We chose cluster FWER<0.2, which was easier to achieve than the traditional FWER<0.05, as we enforced comparable FWER by iteratively lowering the significance threshold, for each of the models (Appendix 2 in the Supplemental Material). The choice of risk alpha does not impact the relative performance of the models, and we can expect models best powered for FWER<0.2 to also be best powered at other FWER levels.

Finally, we reported the proportion of false positive clusters out of all significant clusters (cluster FDR). We labeled false positive clusters, the groups of significantly associated, contiguous vertices that did not contain a TP association.

In follow up analyses, we simulated associations on a single type of vertexwise measurements, to evaluate the probability of false positive (FWER) arising on the same type of measurements, other types of measurements as well as contralateral regions.

#### 2.6.5 Prediction from significant vertices

We evaluated the prediction accuracy achieved from the brain regions reaching significance, in the different mass-univariate models. We used prediction as a meta-criterion to compare the model performances, as it is dependent on power, true and false positives, and association effect sizes. We selected the most significant vertex in each cluster and constructed a linear predictor using association weights [b^i, see Eqs. (1) and (2)] estimated from the different mass-univariate analyses. Because some significant clusters might contain several independent signals, we also built predictors that included all significant vertices. We evaluated the prediction of in the independent UKB and OASIS3 samples.

#### 2.6.6 Mass-univariate analyses of UK Biobank phenotypes

Next, we performed mass-univariate vertexwise analyses on five UKB phenotypes that showed significant replicated morphometricity:^15^ age, sex, BMI, smoking status, and fluid intelligence. We used the raw fluid intelligence score provided by the UKB, a nonstandard test which has demonstrated some reliability in a test–retest analysis.^25^

For each UKB phenotype and model, we reported the number of significant vertices, number of significant clusters as well as their sizes. We defined significance using a Bonferroni significance threshold of $0.05/(652,283\times 5)=1.{5e}^{-8}$, which accounts for the total number of tests performed. For those phenotypes, the true pattern of association is unknown which prevents evaluation of the FPR (or power) of the different approaches. However, false positives or redundant associations should not improve prediction accuracy. In this regard, we evaluated each GLM or LMM model in both the UKB replication and OASIS3 datasets. As above, we used linear predictors and reported the prediction accuracy (correlation) controlling for age, sex, ICV, and site. In OASIS3, we also corrected for clinical status (Alzheimer’s disease and mild cognitive impairment).

## 3 Results

### 3.1 Phenotypes Simulated Under H0

We found that all GLM and LMM models behaved well under the null hypothesis, as indicated by no inflation of test statistic, FPR, or of FPR (FWER). As expected under a stringent Bonferroni correction, all approaches were conservative as indicated by FWER<3% (Fig. S1 in the Supplemental Material).

### 3.2 Phenotypes Simulated Under H1

#### 3.2.1 Inflation of test statistics

**Fig. 2:**
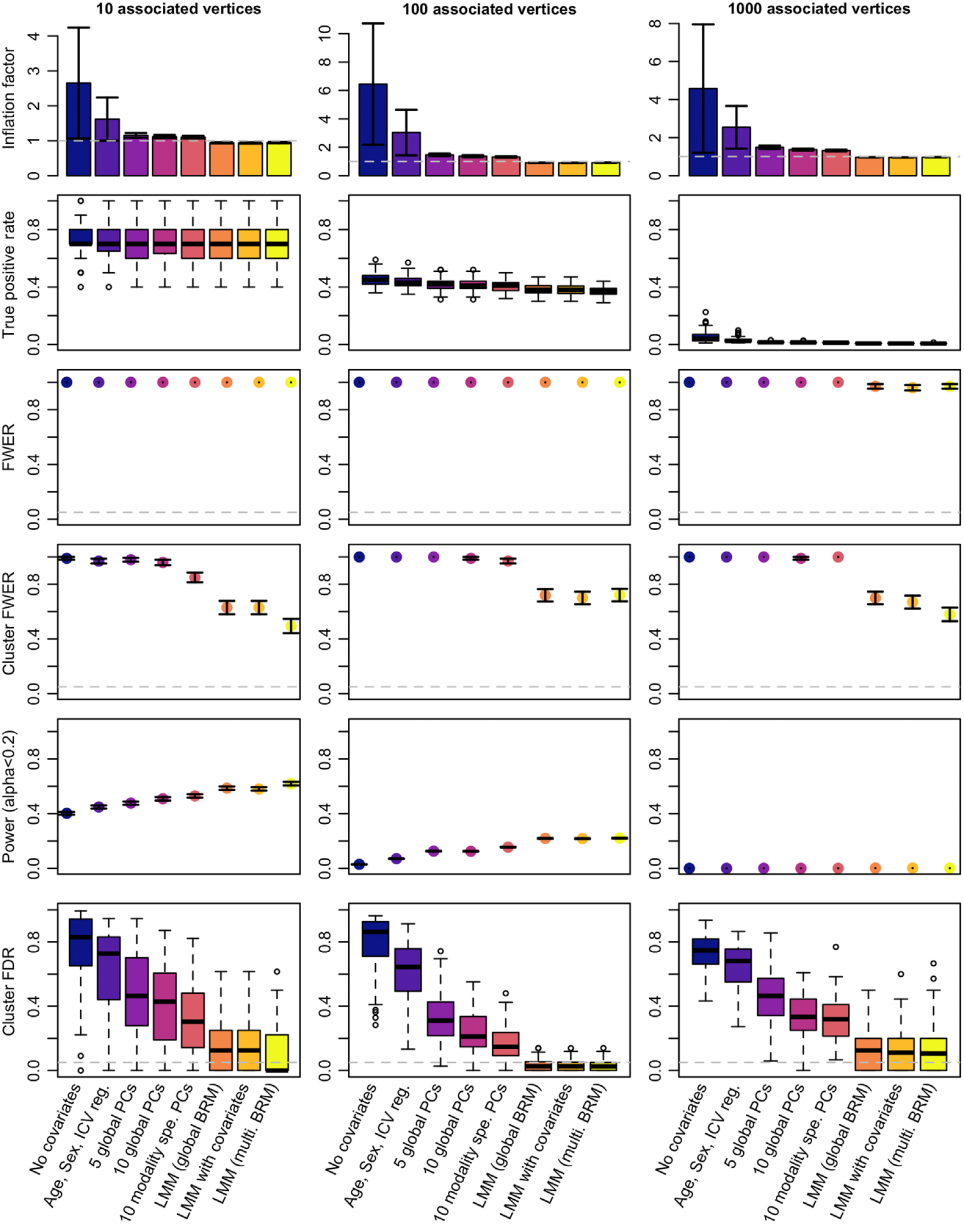
Performance of GLMs and LMMs for mass-univariates vertexwise analyses: test inflation, statistical power, and FPR. The columns correspond to the different scenarios considered when simulating traits. We simulated 100 phenotypic traits for each scenario. Bars represent ±SE across the 100 replicates. Clusters are composed of groups of contiguous vertices each significantly associated with the phenotype (after Bonferroni correction). We labeled them as false positives if they did not include a TP association.

First, we quantified whether we could observe an inflation of test statistics on the vertices not associated with the simulated phenotypes. As expected in presence of correlation between truly associated and null vertices, we observed a global inflation of (median) test statistics when using GLMs (Fig. 2; Table S1 in the Supplemental Material). This was confirmed by an FPR greater than 5% for all GLM models even though controlling for covariates or PCs reduced the inflation of test-statistics compared with the “no covariates” GLM. In comparison, LMMs appropriately controlled the inflation of test statistics on null vertices (λ<1 and FDR<5%; Fig. 2; Table S1 in the Supplemental Material).

#### 3.2.2 True positive rate

First, we confirmed that the TPR (after Bonferroni correction) was dependent on the scenarios that corresponded to different effect sizes for the vertices. For example, about 70% of the truly associated brain regions were detected in the case of a simple trait (10 associated vertices each accounting for 2% of the phenotypic variance on average). On the other hand, <5% of the associated brain regions were identified for the most complex phenotypes (scenario 3, 1000 vertices each accounting for 0.04% of variance, Fig. 2; Table S1 in the Supplemental Material).

Across all scenarios, LMMs exhibited a slightly reduced TPR compared to the GLMs (Fig. 2; Table S1 in the Supplemental Material). We investigated this result using phenotypes simulated from a single type of measurement. We found TPR of LMMs to be especially reduced on subcortical thickness and surface area (Fig. S2 in the Supplemental Material).

#### 3.2.3 False positives

Here, we evaluated the occurrence of false positive vertices or clusters from our simulations. We found that every single simulation yielded at least 1 false positive vertex after Bonferroni correction (FWER=1, Fig. 2). We noted that the FWER of 0.97 (SE=0.02) found for LMMs in the scenario of “1000 associated vertices,” came from three simulations returning no significant associations.

When evaluating the results at a cluster level, we found that using GLMs almost always resulted in one or more false positive cluster (Fig. 2; Table S1 in the Supplemental Material), leading to cluster FWER>85%. Cluster FWER was reduced to 49% to 72% using LMMs (Fig. 2; Table S1 in the Supplemental Material). Despite this improvement, no model ensured a cluster-FWER below 5%. LMMs also minimized the proportion of false positive clusters (cluster FDR), compared with the GLM approaches. At the extreme, more than 70% of the significant clusters were false positives using GLMs without covariate. This reduced to about 60% when controlling for age, sex, and ICV and further reduced to <17% using LMMs (Fig. 2; Table S1 in the Supplemental Material).

Next, we simulated phenotypes associated with a single type of measurement and reported the FWER for each type of measurement in Figs. S3–S6 in the Supplemental Material. This allowed evaluation of whether false positives could appear as a result of associations with vertices from other types of measurements. We found that using GLMs resulted in contamination of signal between all the different types of measurements, as indicated by FWER>5% (Figs. S3–S6 in the Supplemental Material). In comparison, LMMs always minimized the probability of false positives appearing on nonassociated types of measurement. In particular, LMMs ensured that associations on the cortex did not inflate the FPR on subcortical structures, and vice versa (FWER<5%).

#### 3.2.4 Statistical power

We found that the models differ in terms of FPR, which limits the direct comparison of TPR. Instead, we reported the statistical power, which consists of the TPR for a fixed level of FWER (cluster FWER<0.2). We found the LMMs to be more powerful than the GLMs (Fig. 2; Supplementary 2 in the Supplemental Material).

#### 3.2.5 Mapping precision

**Fig. 3:**
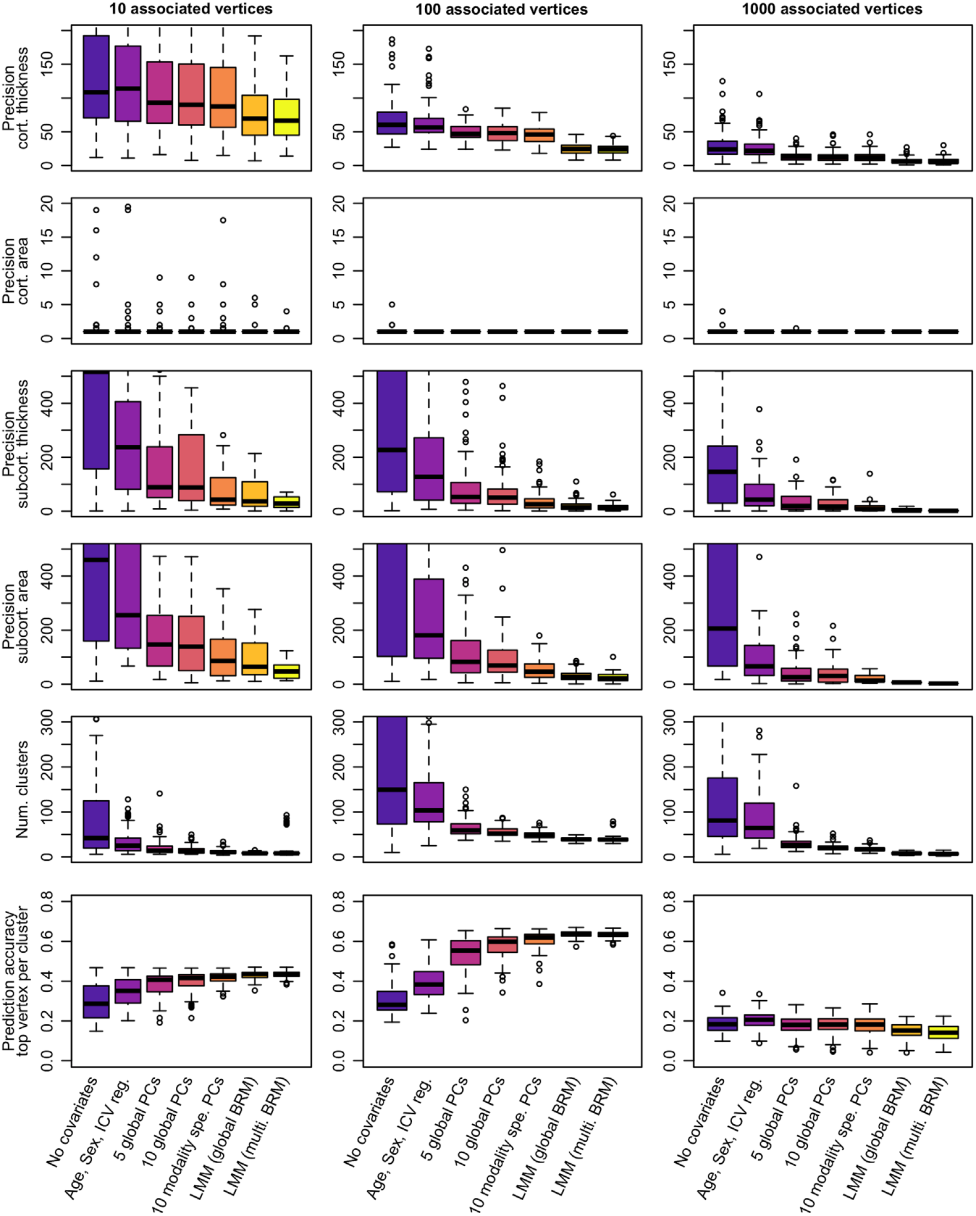
Mapping precision and prediction accuracy from significant vertices between the different models of mass-univariate analyses. The columns correspond to the different simulation scenarios. We simulated 100 phenotypic traits for each scenario. Bars represent ±SE across the 100 replicates. Clusters are composed of groups of contiguous vertices each significantly associated with the phenotype (after Bonferroni correction). We labeled them as TPs if they included a TP association. (Mapping) precision refers to the median size of the TP clusters.

We defined mapping precision as the median size of the TP clusters. LMMs led to a more precise localization of the associations by minimizing the size of TP clusters (whether we looked a clusters median or maximal size, Fig. 3; Table S1 in the Supplemental Material).

The median size of TP clusters was reduced by a factor >10 on subcortical measurements, and by a factor greater than two on cortical thickness when using LMMs (Table S1 in the Supplemental Material). Of note, positive clusters on cortical surface area were particularly small (most clusters were composed of a single vertex), independent of the model used, Fig. 3; Table S1 in the Supplemental Material). However, LMMs still offered a greater precision than the GLMs when considering the maximal cluster size (Table S1 and Figs. S3–S6 in the Supplemental Material).

#### 3.2.6 Prediction accuracy from significant vertices

As a way of aggregating the previous metrics of performance, we compared prediction accuracy achieved from significant vertices, using the UKB replication sample. Across all models and scenarios, selecting the top vertex per significant cluster maximized prediction accuracy, compared with including all significant vertices. This was expected, as significant vertices from the same cluster tag likely redundant information, leading to overweight the prediction signal coming from large clusters.

In simulation scenarios 1 and 2, we found that including more covariates in the GLMs resulted in greater prediction accuracy despite that predictors included fewer vertices (Fig. 3; Table S1 in the Supplemental Material). In addition, LMMs yielded marginally better prediction accuracy than the best GLM using even fewer vertices (Fig. 3; Table S1 in the Supplemental Material), consistent with observation from previous studies.^6^^,^^9^ For the third simulation scenario, the prediction accuracy was comparable and limited for all models (Fig. 3; Table S1 in the Supplemental Material).

#### 3.2.7 Analyses using smoothed cortical surfaces

We repeated the analysis using smoothed cortical meshes of surface and thickness (FWHM = 20 mm), which is more commonly used in the literature than unsmoothed meshes (Tables S4–S8 in the Supplemental Material). We sought to investigate how robust our results were to such variation of MRI processing.

Overall, smoothing did not change the results of the model comparison. LMMs resulted again in a reduced FPR (lower cluster FWER and cluster FDR) as well as reduced power (seemingly more important than in the unsmoothed case). LMMs maximized mapping precision and prediction accuracy, despite relying on fewer significant clusters (Fig. S7 in the Supplemental Material). Of note, performing analyses on smoothed data decreased the mapping precision, leading to TP clusters roughly 10 times larger on cortical meshes (Fig. 2; Fig. S7 in the Supplemental Material).

Data smoothing resulted in a large inflation of test statistic and FPR for GLMs (Fig. 2; Fig. S7 in the Supplemental Material), which is to be expected as smoothing increases the amount of correlation between vertices. We noticed that smoothing led to an increase of cluster FWER for the GLM with 10 PCs, while it decreased cluster FWER for the LMMs (despite the associated vertices and effect sizes remaining the same). This result warrants a more fined-grained evaluation of the associations. We can only hypothesize that the 20 mm (FWHM) smoothing can induce medium-range correlations (hence medium range false positives in GLMs) while it also increases local correlation which might aggregate false positive clusters in LMMs.

### 3.3 Morphometricity of the Phenotypes

First, we confirmed that the morphometricity estimates of our simulated traits matched the values chosen in simulations (Fig. S8 in the Supplemental Material). For the five UKB phenotypes, we also found consistent morphometricity using the three LMM models (Table S2 in the Supplemental Material), suggesting associations across all types of vertex measurements.

BMI and fluid intelligence exhibited large and moderate morphometricity [$R^{2}=0.51$ (SE=0.031) and $R^{2}=0.17$ (SE=0.034)] but only a limited association with age, sex, or the first 10 PCs from vertexwise data (adjusted $R^{2}$ with 10 PCs: $R^{2}=0.032$ for fluid intelligence, $R^{2}=0.033$ for BMI), which resembles the case of our simulations. Age and sex displayed high morphometricity [$R^{2}=0.83$ (SE=0.026) and $R^{2}=0.99$ (SE=0.024)] and large associations with the first 10 PCs (adjusted $R^{2}=0.41$ for age, $R^{2}=0.43$ for sex). Smoking status is a discrete variable (nonsmoker, former smoker, and still smoking) with a morphometricity of $R^{2}=0.12$ (SE=0.029), and adjusted $R^{2}=9.2e-3$ with first 10 PCs (Table S2 in the Supplemental Material). Note that the morphometricity estimates are slightly larger than the ones reported previously,^15^ which had mean cortical thickness and area regressed out.

### 3.4 Analysis of UK Biobank Phenotypes

**Fig. 4:**
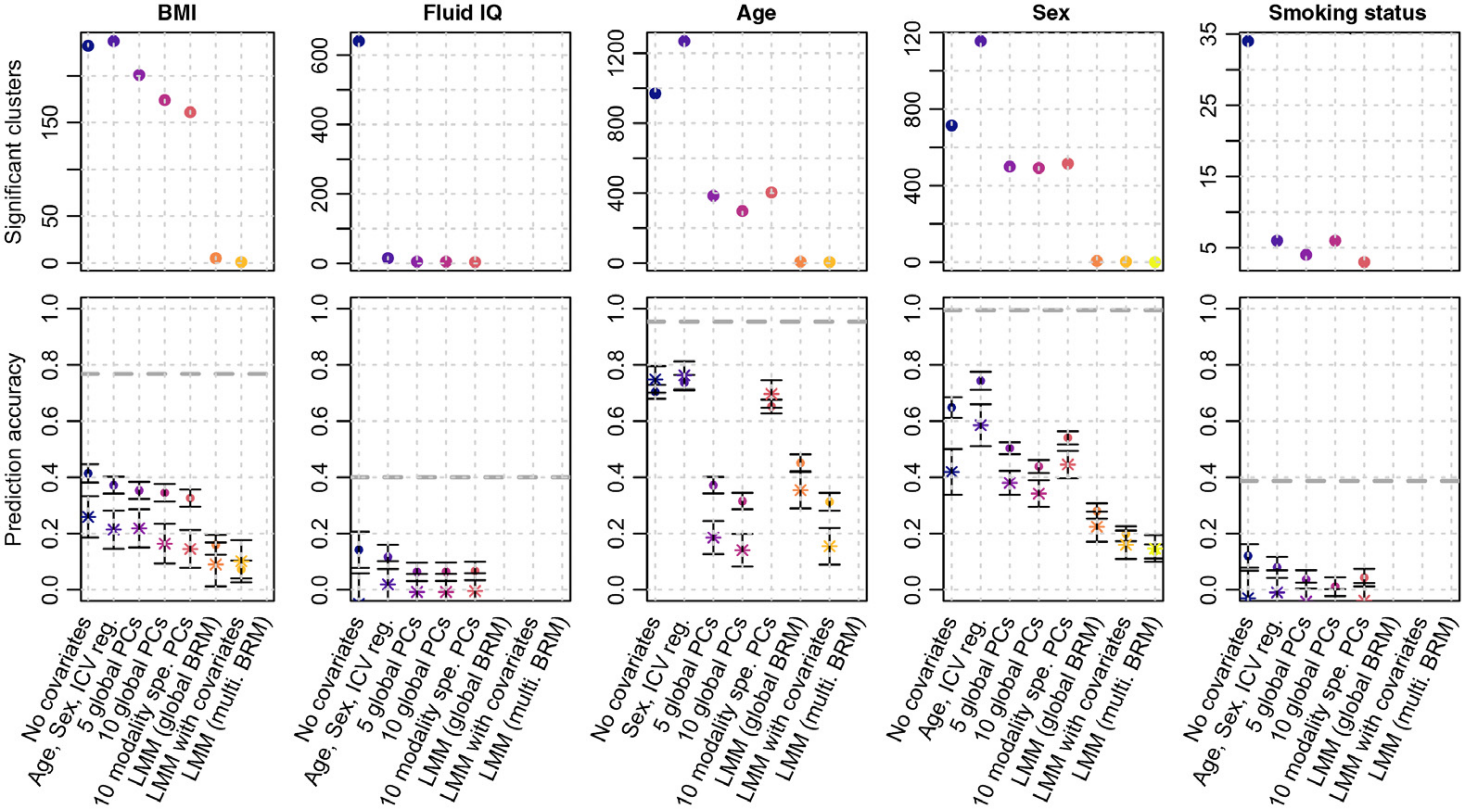
Number of significant clusters and prediction accuracy for the real UKB phenotypes. Bars represent the 95% confidence intervals of the prediction accuracy (correlations). Dots indicate prediction accuracy in the UKB replication sample, while stars correspond to the prediction achieved in the OASIS3 sample. Prediction accuracy is reported controlling for age, sex (when pertinent), ICM, and site/machine. In the OASIS3 dataset, we further controlled for clinical status. The dashed lines correspond to the estimated morphometricity, which corresponds to the theoretical maximum prediction accuracy achievable from a linear predictor.

We sought to confirm the differences in model performance by applying them to real phenotypic traits. Using GLM without covariates resulted in many vertices and clusters reaching significance (Fig. 4; Table S2 in the Supplemental Material). Unsurprisingly, correcting for covariates that account for a large fraction of the phenotypic variance (see adjusted $R^{2}$ with covariates and PCs, Table S2 in the Supplemental Material), drastically reduced the number of associations in the GLMs. For example, correcting for 10 PCs in mass-univariate analyses of age and sex reduced the number of associated vertices by a factor 8 to 13, compared with the GLM without covariates (Fig. 4; Table S2 in the Supplemental Material). For smoking status, the number of significant vertices and clusters also dropped despite a negligible association with PCs (Fig. 4; Table S2 in the Supplemental Material). Similarly, for fluid intelligence, correcting for the top 10 PCs did not remove much of the trait variance over controlling for age sex and ICV (adjusted $R^{2}=0.030$ with age, sex, ICV, adjusted $R^{2}=0.034$ when further controlling for PCs) though it greatly reduced the number of associations. In addition, the more covariates we corrected for, the smaller the size of the associated clusters, suggesting they do remove confounding effects.

We found that across all phenotypes, LMMs resulted in a more parsimonious pattern of associations (Fig. 4; Table S2 in the Supplemental Material). Thus, using the LMM with a single random-effect component, we identified five clusters associated with BMI, 8 with age and 6 with sex (Table S2 in the Supplemental Material). LMM with covariates yielded fewer associations, while LMM with multiple random-effects was the most conservative (Table S2 in the Supplemental Material).

Next, we compared the prediction accuracy achieved from the vertices reaching significance using each model (Fig. 4; Table S2 in the Supplemental Material). Predicting our traits of interest allows evaluation of how power and FPR of the different models may counterbalance each other. In addition, prediction into independent samples quantifies the generalizability of findings obtained in the different mass-univariate approaches. For BMI, we found that prediction accuracy from GLMs in the UKB replication sample was greater than that in the OASIS3 sample, which suggests that GLMs-based predictors capture information that is sample specific (e.g., the same confounders are more likely to be shared in the same cohort than across different cohorts). In contrast, the prediction accuracy from LMMs was comparable between the UKB and OASIS3 samples, pointing toward a better generalizability of the prediction. This suggests that the higher prediction accuracy in the UKB replication sample for GLM is likely to be driven by confounding factors shared between UKB data sets. The comparable performance of GLM and LMM seen on OASIS3 for BMI aligns with our simulations.

For age and sex prediction, prediction accuracy of LMMs was sometimes inferior to that achieved from GLMs, in particular those from the simplest models (“no covariates” and “age, sex, ICV”). Overall, prediction based on LMMs generalized well (comparable accuracy in the UKB and OASIS3), while the GLMs often displayed heterogeneous performances across the test samples (in particular for the GLMs with PCs, which may suffer from PCs being different between samples).

Regarding fluid IQ and smoking status, no LMM predictor was available, and the different GLMs resulted in comparable, albeit limited prediction accuracy.

### 3.5 Description of Associated Regions

We listed the significant associations identified using LMM (global BRM) in Table S3 in the Supplemental Material (Figs. S9–S11 in the Supplemental Material for Manhattan plots, and Figs. S12–S16 for brain plots in the Supplemental Material). The significant associations were in the range of $R^{2}=0.5\%$ to 1%. Most associations were observed with subcortical volumes though the top cluster for sex was spatially located at the border of the lateral–orbitofrontal and medial orbitofrontal gyri (based on the Desikan atlas^26^). Out of the 85 vertices associated with age, sex, and BMI, 68 replicated in an independent UKB sample (p<0.05/85, Table S2 in the Supplemental Material). In particular, 4/11 associations replicated for BMI, 43/47 for age, and 21/27 for sex. The replication rate was slightly lower in the OASIS3 dataset, where none of the vertices reached significance for BMI, 15/47 associations were replicated for age, and 12/27 for sex. Overall, the sign of the associations was consistent across the three datasets (Table S3 in the Supplemental Material).

## 4 Discussion

Using extensive and realistic simulations, we evaluated the statistical power, FPR, and precision of GLMs and LMMs for vertex-wise gray-matter association studies. In particular, we evaluated the different models in the context of big-data neuroimaging (large sample size but even greater number of correlated brain vertices).^27^ We consistently found that using state-of-the-art GLMs resulted in a large number of false positive associations and clusters, whether we used smoothed or not-smoothed gray-matter surfaces. Thus, across all scenarios tested, more than 60% of the significant clusters were false positives using a standard GLM that controlled for age, sex, and ICV. In comparison, FDR was below 17% using LMMs, though still greater than the 5% expectation (Table S1 in the Supplemental Material, Fig. 2, and Fig. S7 in the Supplemental Material). In addition, we showed that unlike GLMs, LMMs could appropriately separate cortical from subcortical associations, even though signal contamination between thickness and surface still occurred (Figs. S2–S5 in the Supplemental Material).

Our results suggest that previously reported results from mass univariate vertexwise analyses obtained using standard GLM approaches could contain many redundant associations, some of which are likely to be false positives induced by confounding factors that cause correlation between vertices [e.g., Refs. 28 to 31, see also Fig. 1(b)]. Note that albeit redundant in term of association and prediction, some of the brain regions identified using GLM may correspond to indirect manifestations of the trait/disease of interest, which may be relevant to understand the dynamics of gray-matter structure. Importantly, the type 1 error (>5%) we observed in simulations also warns against taking for granted results from LMMs.

The increased FPR for GLMs has been well documented in omics association analyses studies (e.g., GWAS^8^^,^^14^ or MWAS^6^^,^^9^) and has been attributed to proximal and distal correlations between features, caused by factors independent of the trait of interest (e.g., genetic ancestry in genetics,^14^ cell composition of the biological sample, and smoking status in DNA methylation^6^^,^^32^). On the other hand, LMMs can reduce the probability of generating false positives, by fitting all other vertices as random effects which accounts for the complex correlation structure between vertices within and between individuals. In brain imaging, more work is needed to identify the factors that contribute to local and distal correlations between vertices, hence inducing a correlation between true associations and “null” vertices, beyond the usual covariates or confounders used in neuroimaging (e.g., MRI scanner/artefact^1^ or demographics^2^).

LMMs yielded fewer TP associations, using the Bonferroni adjusted significance threshold (in particular for the simulated associations on the subcortical nuclei; Fig. S2 in the Supplemental Material). However, this result must be interpreted with caution as it may be partly due to a more stringent control of false positives, resulting in overall fewer vertices reach significance (Fig. 2; Table S2 in the Supplemental Material). To better compare the models performances, we estimated statistical power (i.e., TPR for a set FPR) and noted that the LMMs were more powerful than the GLMs (Fig. 2, Table S2 in the Supplemental Material, Supplementary 2 in the Supplemental Material).

Despite this, LMMs are known to suffer from a power reduction, which arises from the double fitting of the vertex of interest, once as fixed effect and again as a random effect [Eq. (2)].^7^^,^^33^ For subcortical structures, the effect of double-fitting could be exacerbated by the high level of correlation between vertices. A workaround^7^ is to exclude the candidate vertex (and vertices strongly correlated) from the BRM calculation,^33^ though this requires computation of the BRM p times (complexity is $O(pN^{3})$, where N is the sample size and p is the number of vertices), which becomes impractical for large sample sizes.^7^^,^^33^ In comparison, the current LMM implementation makes our analysis scalable to samples sizes of tens of thousands [computational complexity of $O(pN^{2}+N^{3}+pN)$].^6^ It should be noted that restricted maximum likelihood estimation approach used in LMMs requires substantially more computational resources than the GLMs and thus requires the use of high-performance clusters.

Beyond power and FPR, we observed from simulations that LMMs could pinpoint the gray-matter association with greater precision (smaller clusters of TPs, Fig. 3). Lastly, we found that prediction achieved from clusters reaching significance in LMMs was on par with that from the best GLMs (Fig. 3), despite fewer vertices included in the predictor. This suggests a higher specificity of the LMMs. Overall, our simulations indicate that LMM with a single random effect currently offers a good trade-off between power and FPR. However, it still fails to ensure a cluster FWER below 5% (also reported on MWAS^6^), despite a stringent Bonferroni correction to account for multiple testing.

Next, we applied the mass-univariate vertexwise models to five real phenotypes of the UKB: age, sex, BMI, smoking status, and fluid IQ. As in the simulations, the LMMs identified fewer vertices and clusters than the GLMs (Fig. 4, Table S2 in the Supplemental Material). The LMM with multiple random-effect components was the most stringent (a single cluster of association), consistent with simulations which showed it had the lowest FWER and statistical power. In contrast, the LMM with a single random-effect component identified several cortical and subcortical associations with BMI, age and sex (Table S3 in the Supplemental Material). Most (12/19) of the top vertices in the associated cluster replicated in the UKB left out sample, and six replicated in the OASIS3 sample (Table S3 in the Supplemental Material). The lower replication rate in OASIS3 may be due to a lower power even though we cannot rule out that the same confounders might act similarly on the two UKB data sets. Overall, replication may be warranted to conclude about an association in future studies, considering the inflation of false positives (even when using LMMs, Fig. 2). The top associated vertices with age, sex, and BMI each captured <1% of the phenotypic variance, suggesting that many more small associations are likely to account for the full morphometricity of the phenotypes (Table S2 in the Supplemental Material). Our results echo the warning against the risk of small associations being confounded (e.g., by artifacts) in big-data neuroimaging,^27^ which was confirmed by a recent exploratory study of putative MRI confounders in the UKB.^3^ Note that LMMs can reduce false positive associations caused by correlations across and within the different types of measurements (Figs. 2 and 3). Finally, unlike in our simulations (Fig. 3), LMMs often resulted in lower prediction accuracy than GLMs in the UKB left out sample (Fig. 4). Nonetheless, prediction from LMMs generalized better in the OASIS3 dataset (Fig. 4).^24^ This suggests that LMMs result in a more robust and parsimonious predictor, less sensitive to sample specific vertexwise patterns and confounders.

In the past years, many studies have been published on the association between gray-matter structure and our phenotypes of interest (see Tables S4–S8 in the Supplemental Material for a selective review of publications). Our simulation and empirical results suggest that some of these studies could report a substantial number of false positive or redundant associations. Nevertheless, due to the limitations outlined below, it is unclear which of these studies suffer from this issue and to which extent.

First, it has been shown in the omics literature that power of LMM may be reduced for phenotypes strongly associated with the covariation between features.^7^^,^^34^ This is likely the case for age and sex as indicated by their strong association with the PCs calculated from vertex–wise data (Table S2 in the Supplemental Material). This may be an important limitation for phenotypes associated with a cascade of changes in gray-matter, for which LMM would be over conservative.

In addition, LMM assumes a normal distribution of random effects, which may not be realistic for all phenotypes studied. It is equivalent to assuming highly regionalized and specialized brain regions, each displaying a small association with the phenotype. Thus, LMM may be suboptimal under some architectures of association, such as if only a specific but sizable brain region is associated with the trait. Several models have been proposed to relax the LMM hypothesis, for example, to include large/outlying associations as fixed effects (stepwise LMM^35^), break down the feature list into sets of small and large associations (data driven approach: MOMENT^6^), or consider more complex distributions using Bayesian LMMs (Bayesian alphabet^34^^,^^36^). They remain to be evaluated in the context of vertexwise analyses. More simulations are warranted, to study other trait architectures, different trait distributions (e.g., skewed, discrete) or to evaluate more sophisticated models. Of note, we limited our trait complexity to 1000 associated brain regions, even if the true pattern of association might be more complex. Our simulations suggest that LMM outperform a GLM independently of the trait complexity, but also that larger samples are required to study traits with more complex architecture (Fig. 2). Our framework of simulation may be easily adapted for such investigations and offers the advantage of estimation of statistical power as well as FPR, which are not often reported at the same time.^37^^,^^38^

The nature of the gray-matter regions identified in our GLM analyses of real phenotypes (for which the truth is unknown) can be a matter of debate, which depends on the (also unknown) nature of the correlation between vertices. Two key scenarios can explain the correlation but the data currently available to us does not allow to differentiate between them. First, the correlation could be solely due to confounders [e.g., Fig. 1(b)], in which case the distal associations are false positives. Second, the correlation between vertices could reflect dynamic brain pathways relevant to the trait of interest. In this case, one could describe the GLM associations not found using LMM as redundant rather than false positives. Since we cannot differentiate between these two important causes of between-vertex correlation, we chose to label LMM models as parsimonious, until we understand better the effect of confounders on the vertices correlation structure as well as the longitudinal changes in gray-matter and their relationship with the phenotypes.

Finally, some additional limitations are worthy of note as they may limit the interpretation of mass-univariate vertexwise analyses (compared with GWAS results). First, gray-matter associations may be both causes or consequences of the phenotype studied, unlike GWAS findings, which can impact how to consider redundant associations. At one end of the spectrum are phenotypes such as age for which the direction of the causality is obvious (nothing causes chronological age). When describing which parts of the brain are affected by aging, one may be interested in reporting all associations, including all indirect and redundant. Though, there is no guarantee that those brain regions correctly map the brain pathway of ageing as they might also reach significance due to confusion factors. On the other hand, for many other phenotypes, the direction of causality is unclear (e.g., smoking, BMI) and one may prefer a more parsimonious and robust brain mapping. Second, gray-matter vertices are semiarbitrary features which may be defined and measured in different ways (e.g., different cortical meshes in FreeSurfer). For instance, the resolution of the cortical tessellation is arbitrary and thus so is the number of local vertices which are found to be significant. Hence, the results presented might differ if one were to use a different MRI processing or vertex definition (e.g., volume processing from SPM, coarser surface mesh). In addition, we used Bonferroni to control for multiple testing, although approaches based on RFT are more commonly used (Tables S4–S8 in the Supplemental Material).^16^ RFT-based correction is reportedly less stringent than Bonferroni (at least for smoothed data, on which the RFT hypotheses are more likely to be met),^39^ which suggests that RFT would also suffer from the inflation of test statistics that we reported.

Furthermore, we did not consider all possible covariates in GLM analysis, focusing on the more commonly used in previous analyses (age, sex, ICV, Tables S4–S8 in the Supplemental Material). More work is needed to evaluate the extended set(s) of covariates which have been recently proposed, from a large-scale study of the UKB data.^3^ Finally, mass-univariate results may depend on the study sample used, which raises the question of generalizability into to samples from different age or ethnic groups or with different MRI qualities for instance.

In summary, we found that results obtained using the current state-of-the-art models (GLMs) used in MRI-trait association analyses likely suffer from a large inflation of false positive or redundant associations due to the unaccounted correlation between vertices. In contrast, LMMs allow to control for all vertices fitted as a random effect, which result in a more parsimonious, robust, and conservative characterization of the localized associations between a phenotype and gray-matter structure. However, LMM results should still be interpreted with caution as our simulations show that the FPR remains higher than the standard type 1 error of 5%, even after Bonferroni correction.

## Code, Data, and Materials Availability

Data used in this manuscript are held and distributed by the OASIS and UKB teams. We have released the code used in image processing and analyses to facilitate replication and dissemination of the results (https://baptistecd.github.io/Brain-Mapping-LMM/). Upon publication, we will also release, the summary statistics of mass-univariate analyses performed on the UKB phenotypes (https://cnsgenomics.com/content/data). Supplementary figures (Figs. S1–S16), tables (Tables S1–S7), and sections (Supplementary 1 and 2) may be found on the GitHub repository: https://github.com/baptisteCD/Brain-Mapping-LMM/blob/main/Supp._Article_JMI.pdf. Note: JMI does not require review of supplemental material; supplemental material may not have been reviewed.
