# Supplementary material for: Parsimonious model for mass-univariate vertexwise analysis
Source: J Med Imaging (Bellingham). 2022 May 20;9(5):052404. doi: 10.1117/1.JMI.9.5.052404 (PMC9122091; doi:10.1117/1.JMI.9.5.052404)
Supplement: Supplementary file 1 [file JMI_009_052404_SD001.pdf]

Supplementary Information for: **A parsimonious model for mass-univariate vertex-wise analyses**

Baptiste Couvy-Duchesne, Futao Zhang, Kathryn E. Kemper, Julia Sidorenko, Naomi R. Wray, Peter M. Visscher, Olivier Colliot, Jian Yang

Correspondence: BCD (b.couvyduchesne@uq.edu.au), JY (jian.yang@uq.edu.au)

This PDF file includes:

Supplementary Figs. 1 to 16
Supplementary Tables 1 to 7
Supplementary (section) 1 - 2

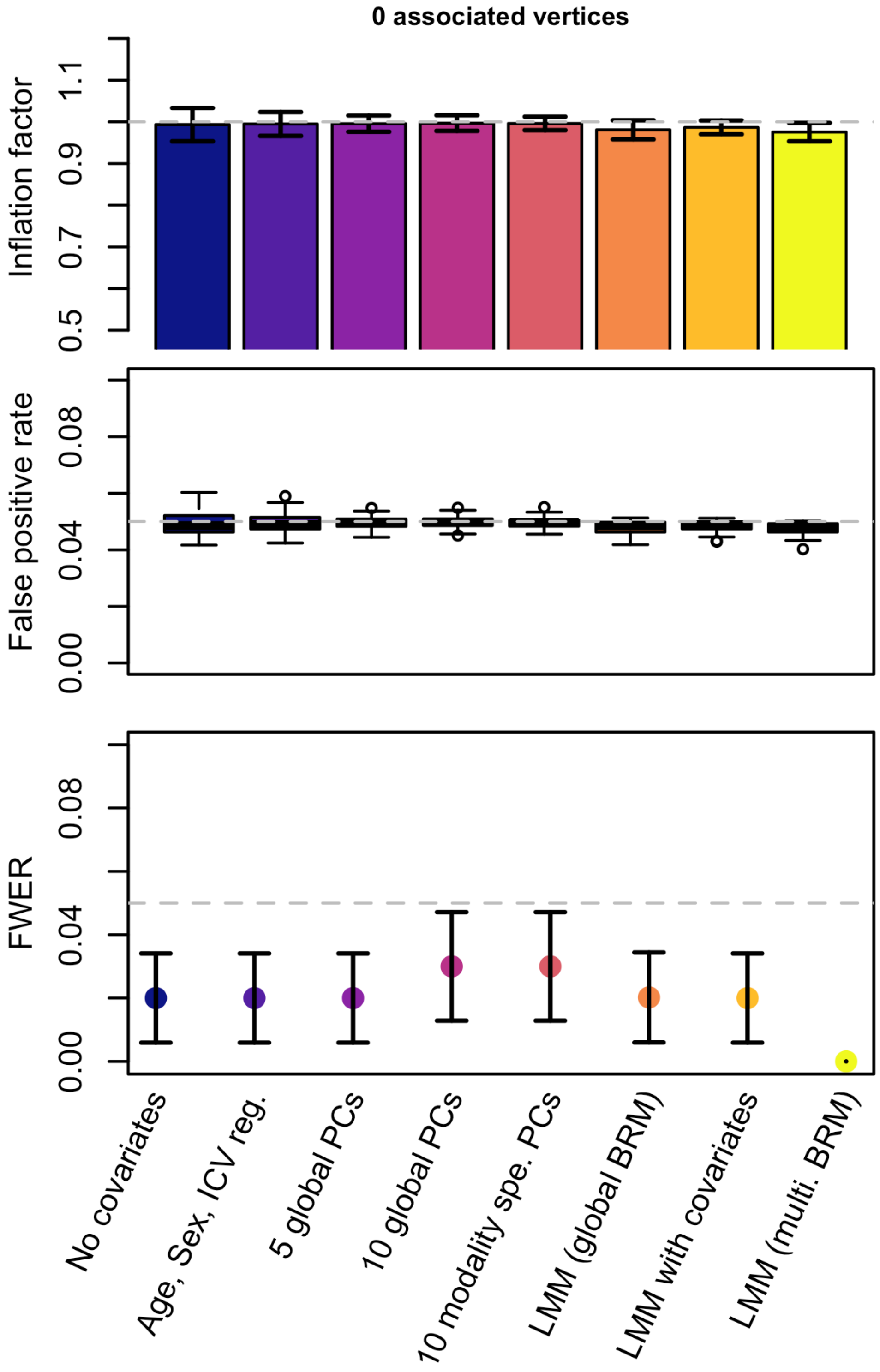

SFigure 1: Summary of mass-univariate analyses on null phenotypes (not associated)

A hundred phenotypes were simulated at random and analysed using each model.

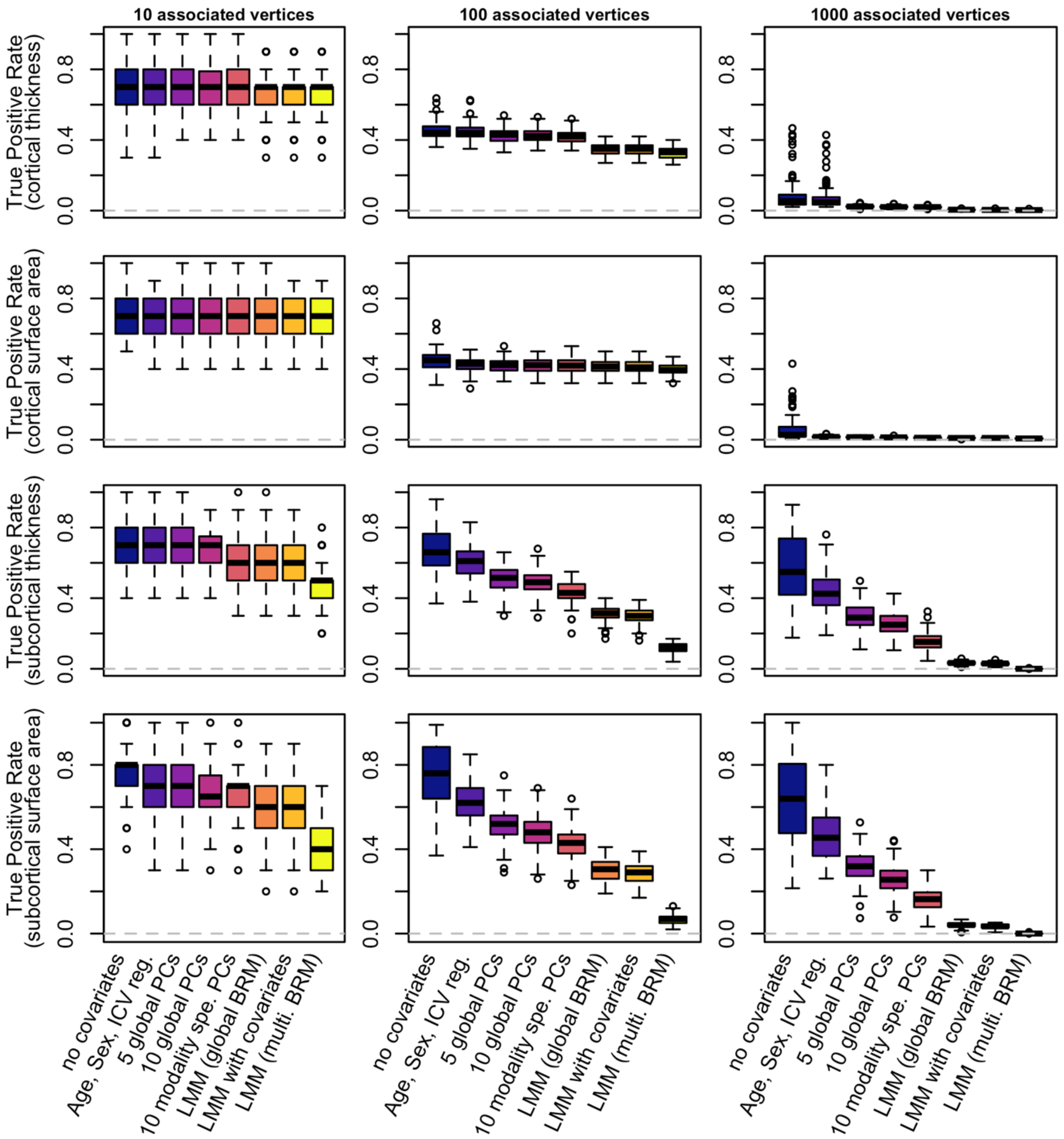

SFigure 2: Statistical power for simulated phenotypes associated with a single type of modality

The phenotypes were simulated from a single type of measurement (indicated in parenthesis on the Y-axis label). The statistical power is measured using the true positive rate, or proportion of true associations significant after Bonferroni correction.

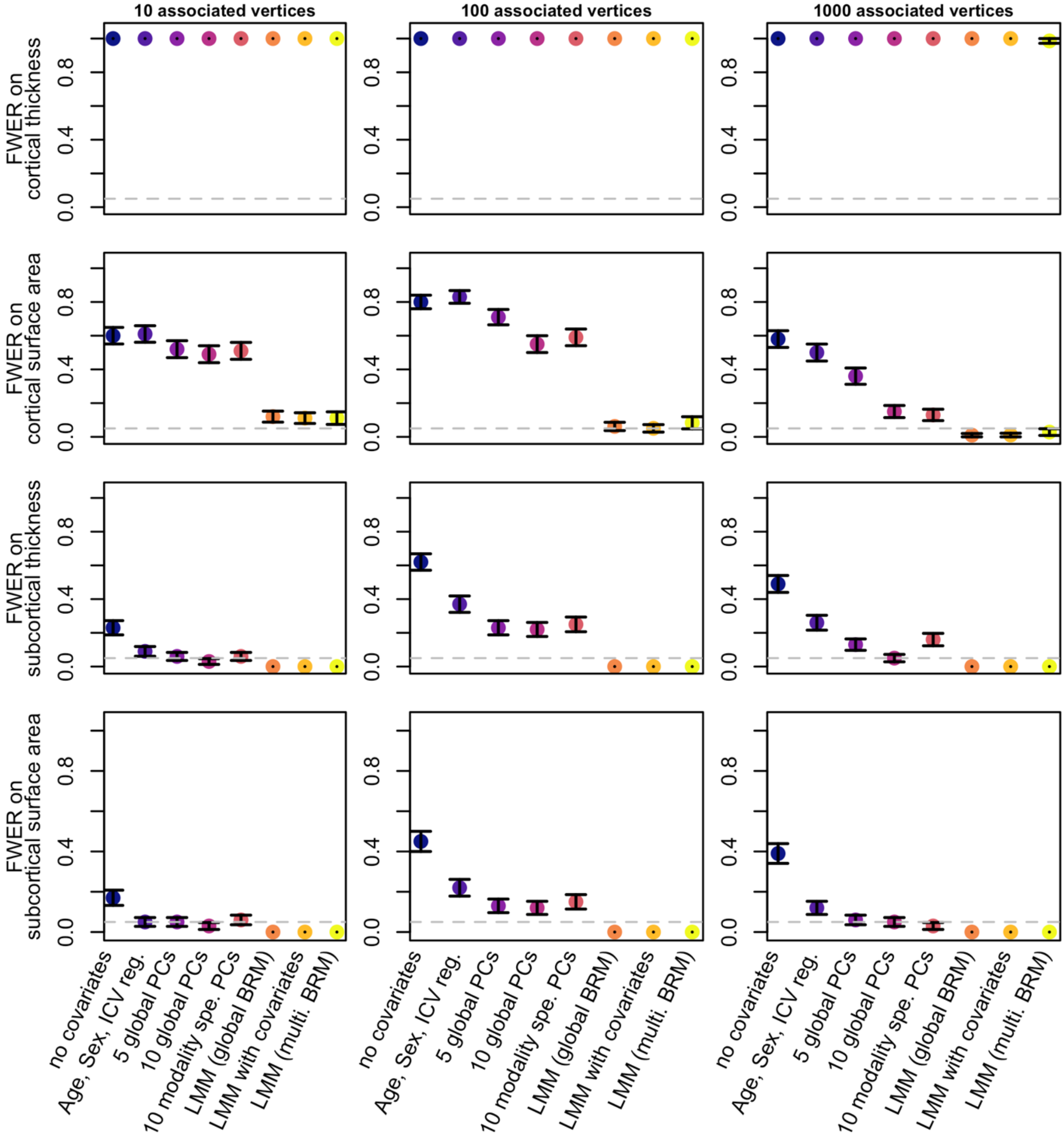

SFigure 3: Probability of a false positive vertex arising from associations on cortical thickness

The phenotypes were simulated from cortical thickness only. The FWER corresponds to the proportion of replicates with a false positive vertex on each type of measurement.

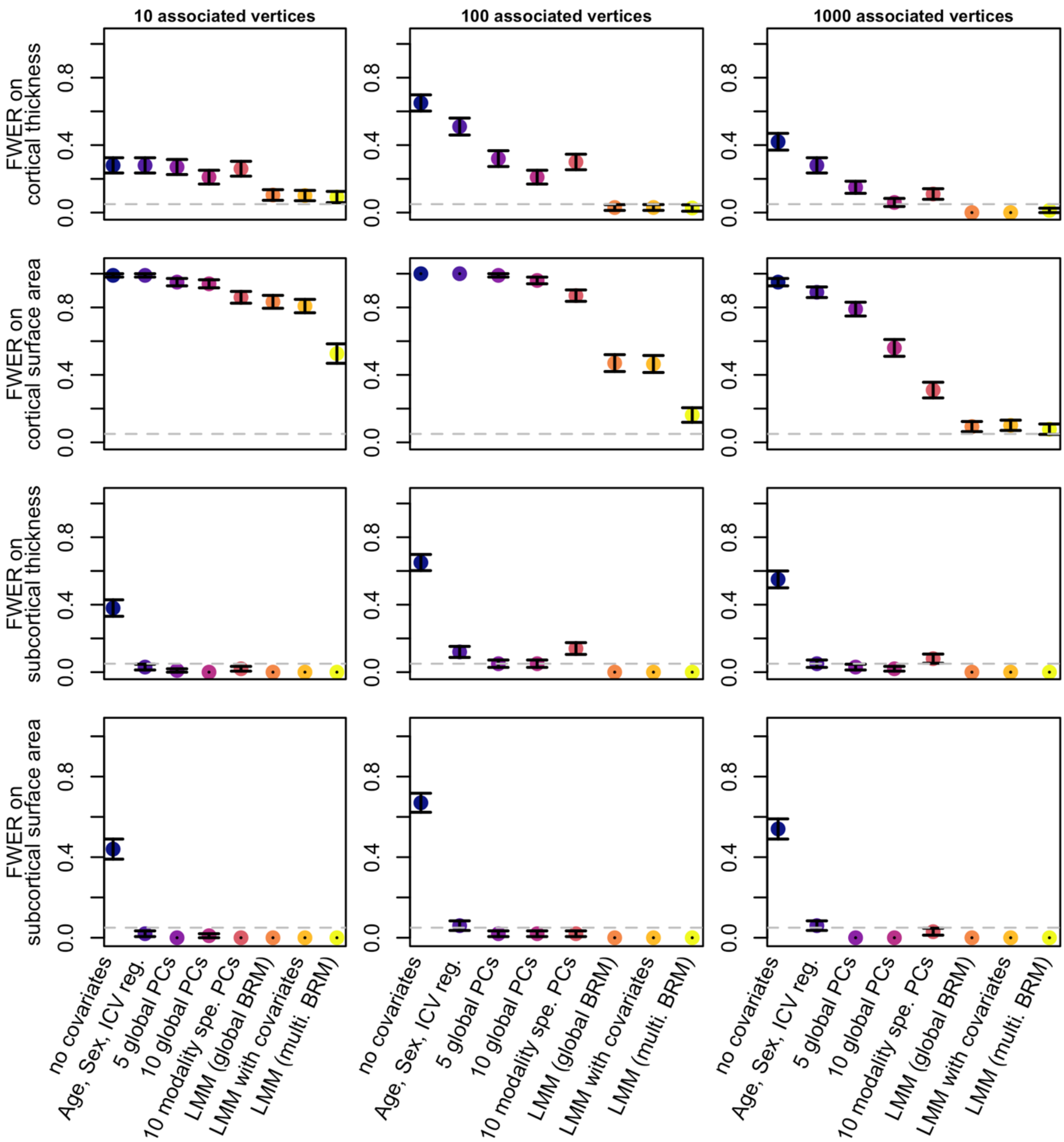

SFigure 4: Probability of a false positive vertex arising from associations on cortical surface area

The phenotypes were simulated from cortical surface area only. The FWER corresponds to the proportion of replicates with a false positive vertex on each type of measurement.

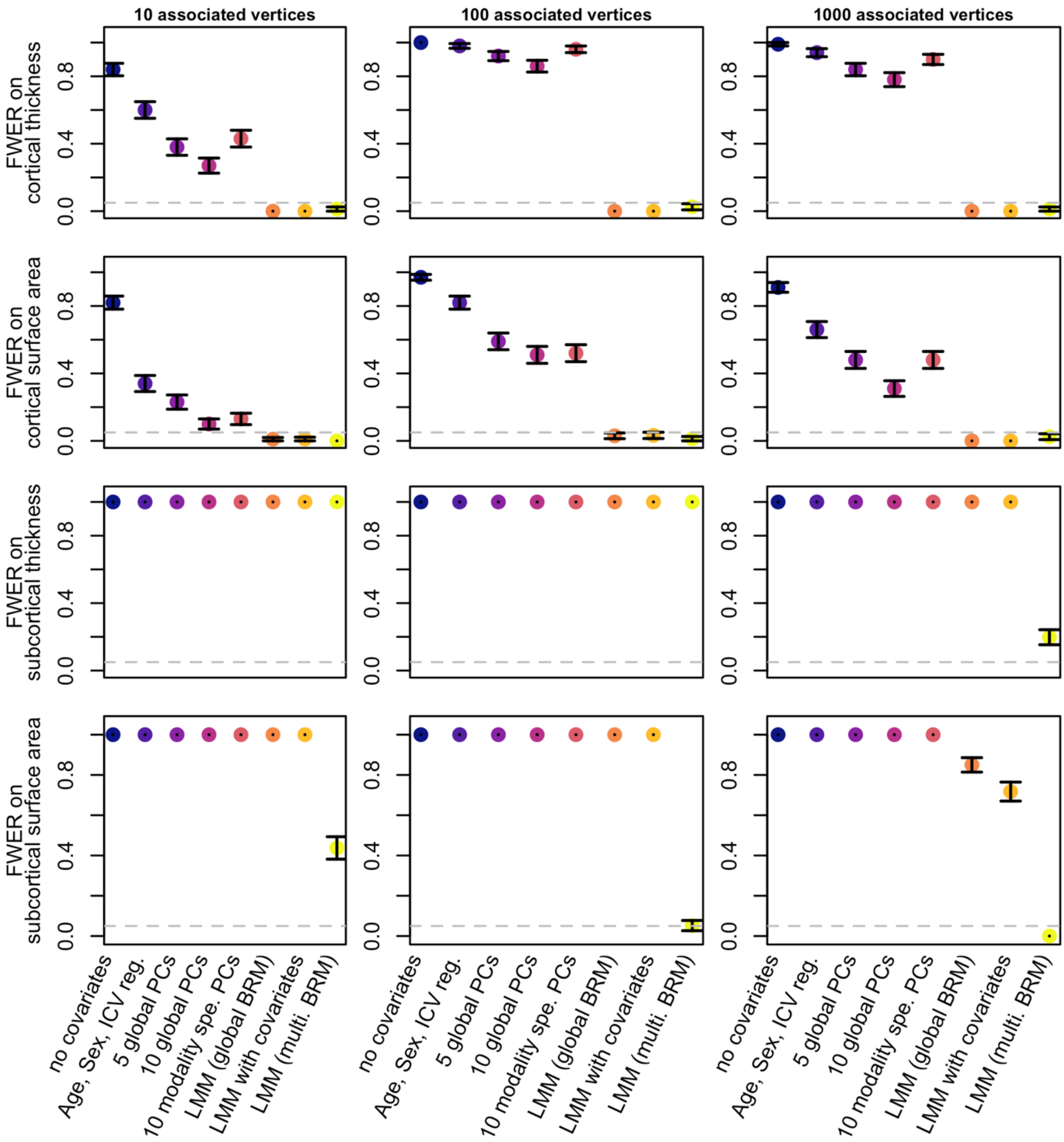

SFigure 5: Probability of a false positive vertex arising from associations on subcortical thickness

The phenotypes were simulated from subcortical thickness only. The FWER corresponds to the proportion of replicates with a false positive vertex on each type of measurement.

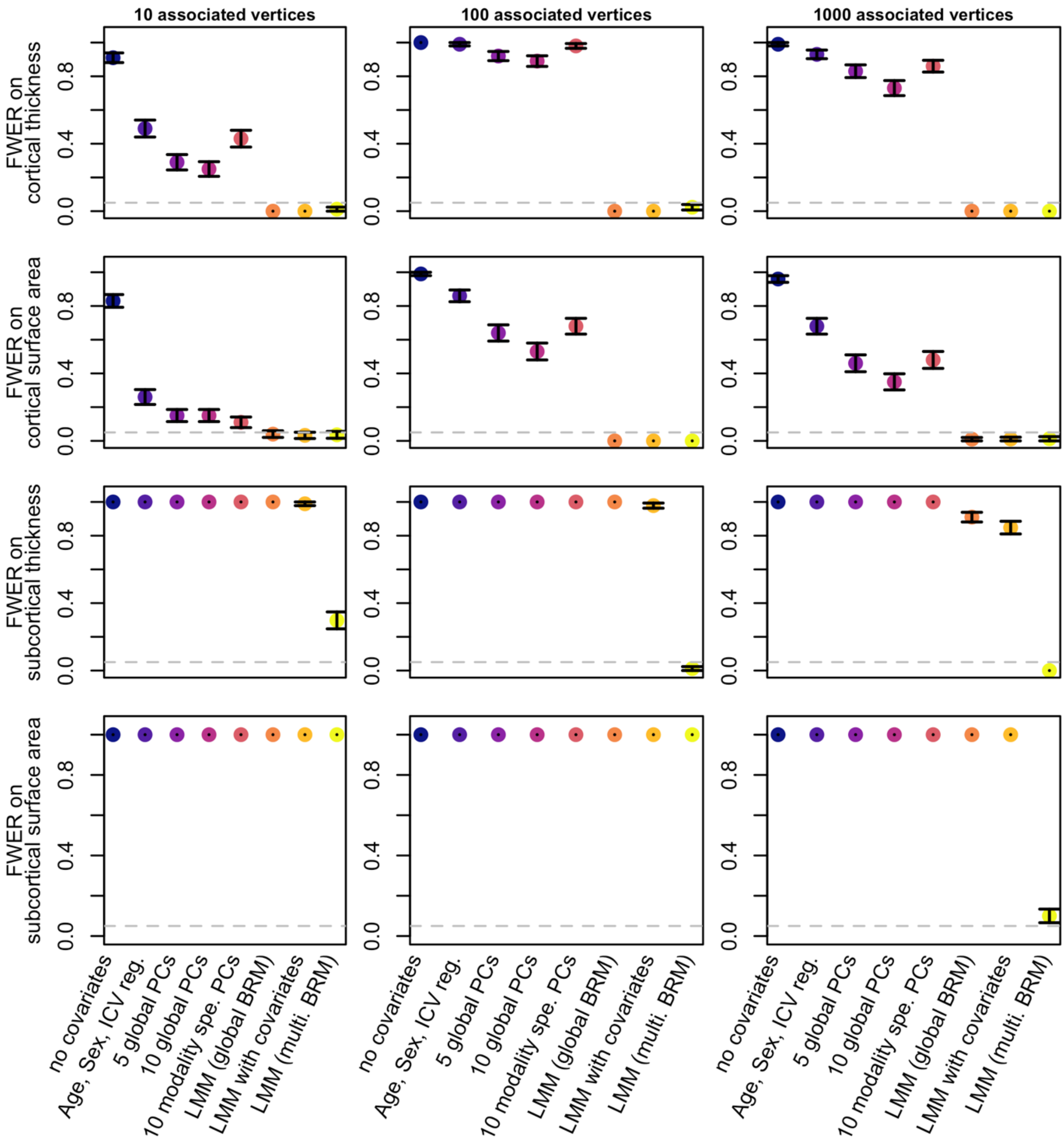

SFigure 6: Probability of a false positive vertex arising from associations on subcortical surface area

The phenotypes were simulated from subcortical surface area only. The FWER corresponds to the proportion of replicates with a false positive vertex on each type of measurement.

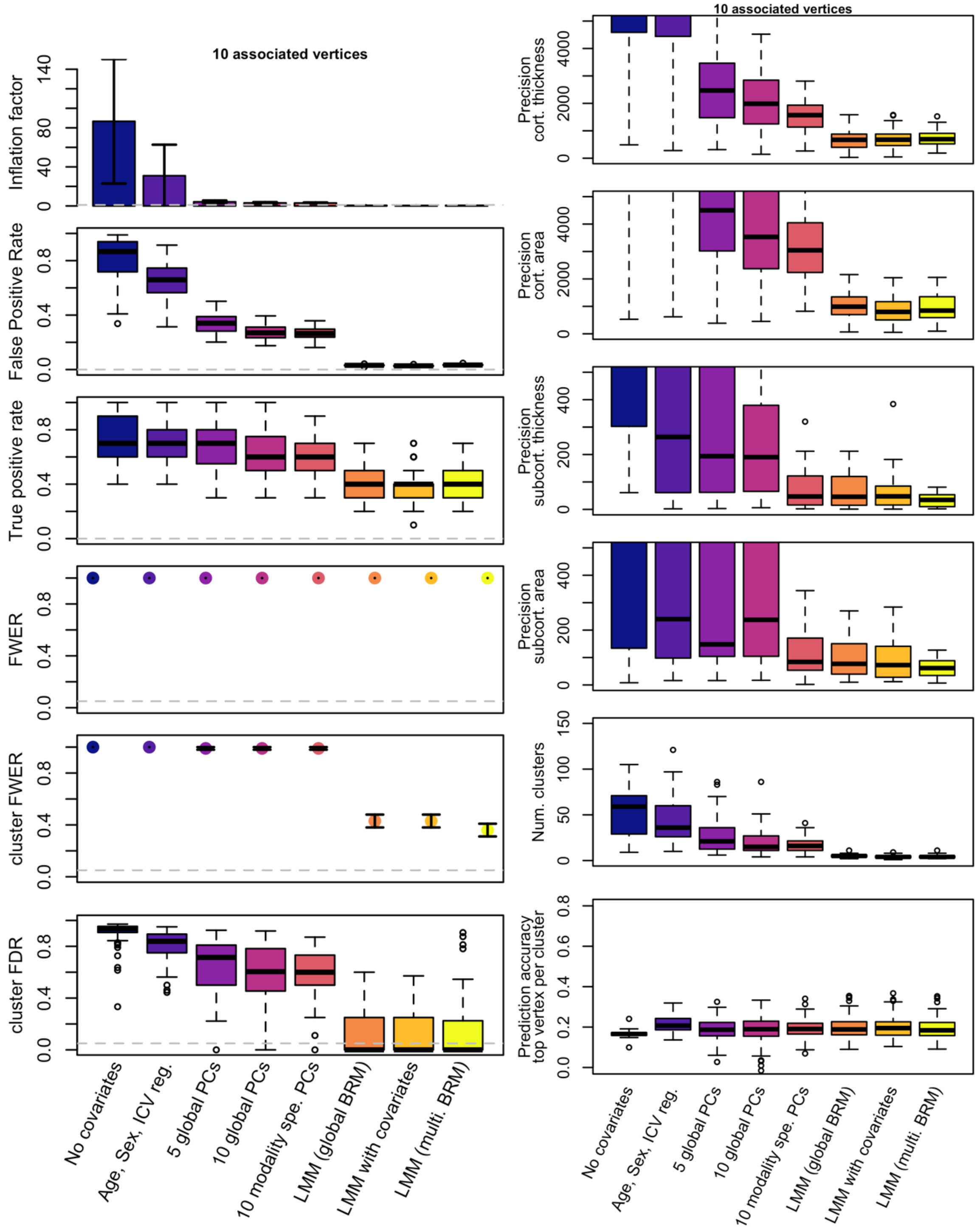

SFigure 7: Results of mass-univariate analyses using smoothed meshes of cortical vertices.

For the ease of computation, we only considered a simple simulation scenario of 10 associated vertices (morphometricity of 20%). Phenotypes were again simulated from the cortical and subcortical meshes, using the same seed vertices and weights as in previous simulations on unsmoothed data.

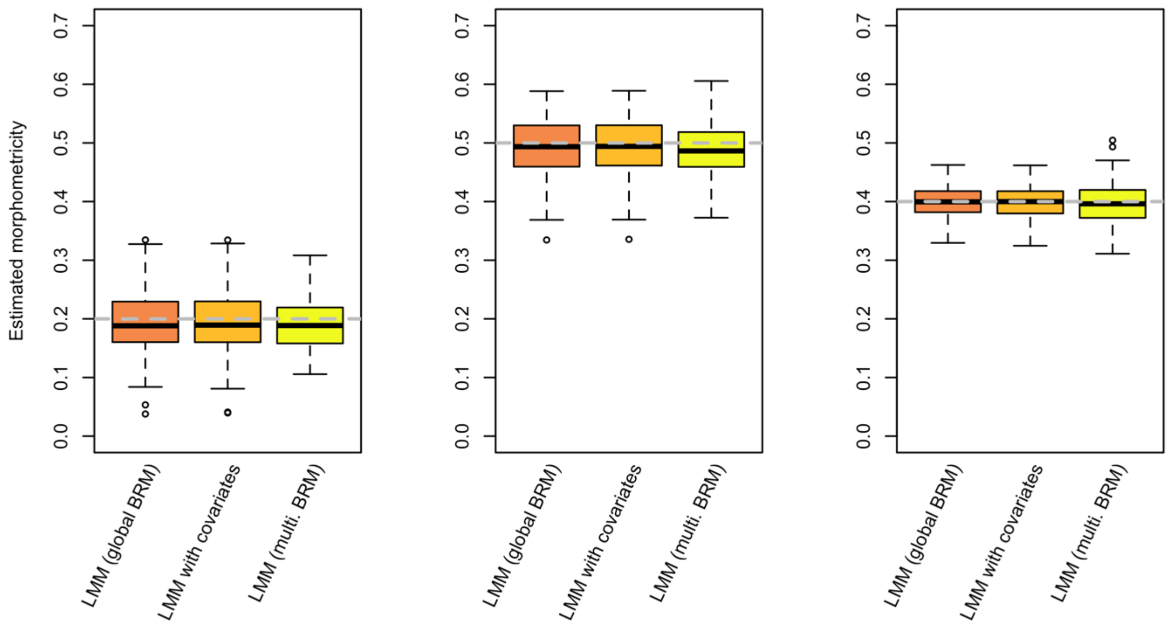

SFigure 8: Morphometricity estimates of our simulated traits from our three LMM models
The grey lines represent the expected values. The different panels correspond to the different simulation scenarios : “10 associated vertices”, “100 associated vertices” and “1000 associated vertices”.

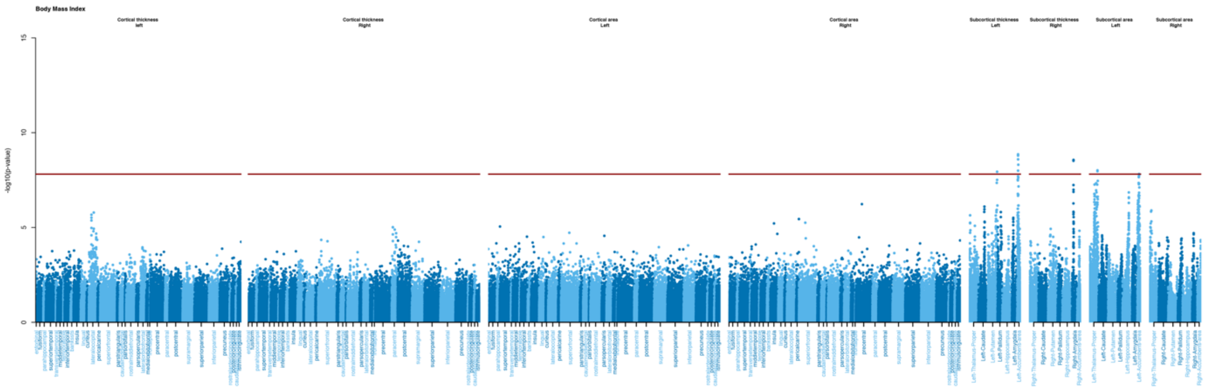

SFigure 9: Manhattan plot of mass-univariate vertex-wise analysis of body mass index

The Y axis shows the significance level (-log10 pvalue) for all grey matter vertices using the LMM “single random effect”. The red horizontal line indicates the brain-wide significance threshold using Bonferroni correction.

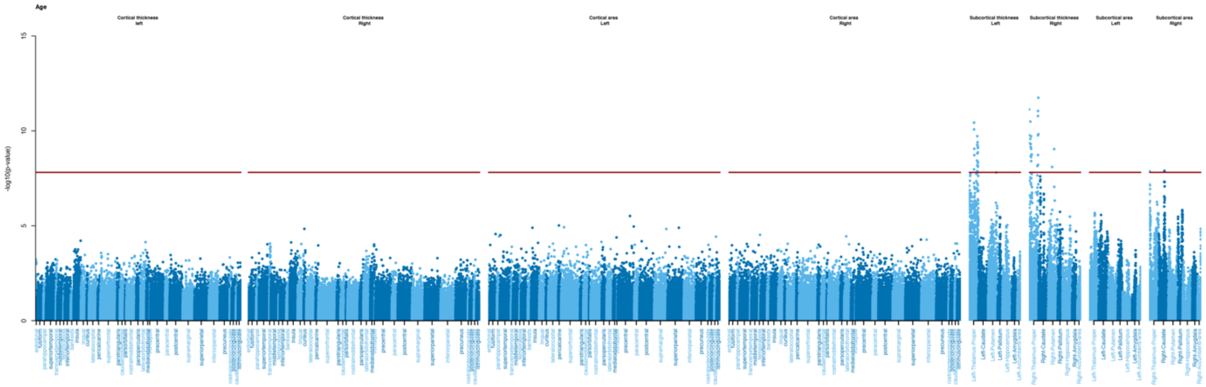

SFigure 10: Manhattan plot of mass-univariate vertex-wise analysis of age

The Y axis shows the significance level (-log10 pvalue) for all grey matter vertices using the LMM “single random effect”. The red horizontal line indicates the brain-wide significance threshold using Bonferroni correction.

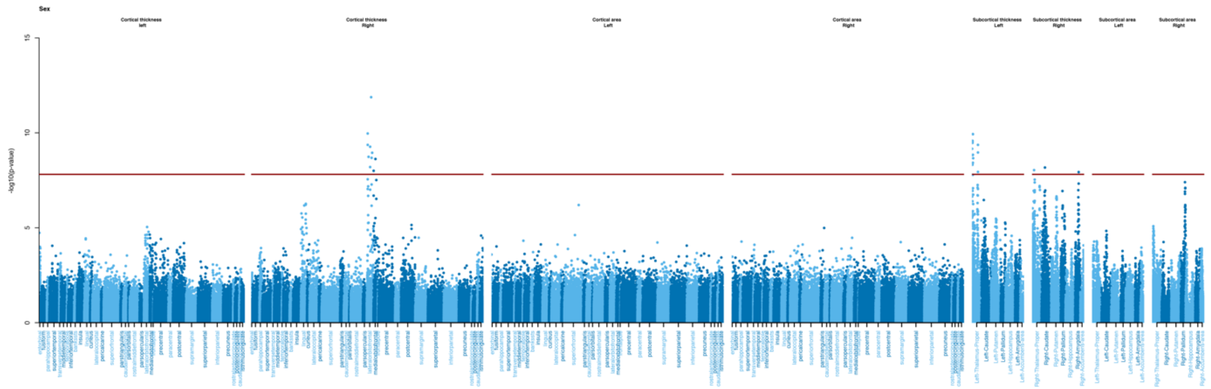

SFigure 11: Manhattan plot of LMM mass-univariate vertex-wise analysis of sex

The Y axis shows the significance level (-log10 pvalue) for all grey matter vertices using the LMM “single random effect”. The red horizontal line indicates the brain-wide significance threshold using Bonferroni correction.

a)

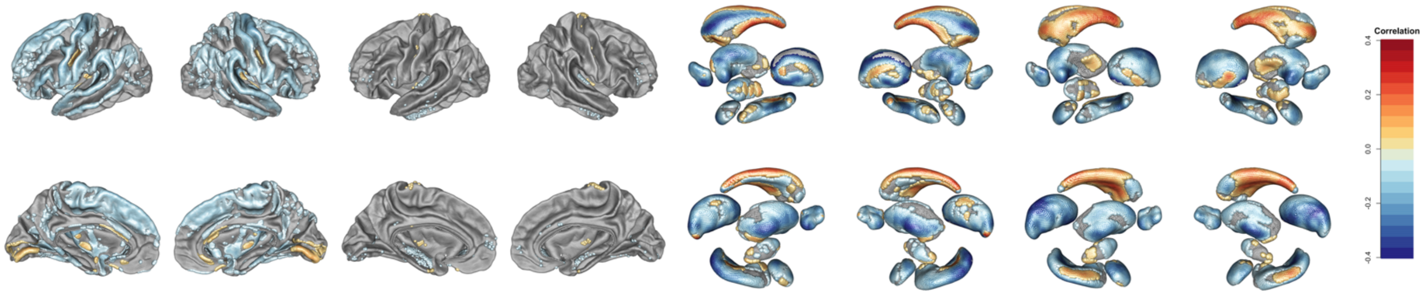

b)

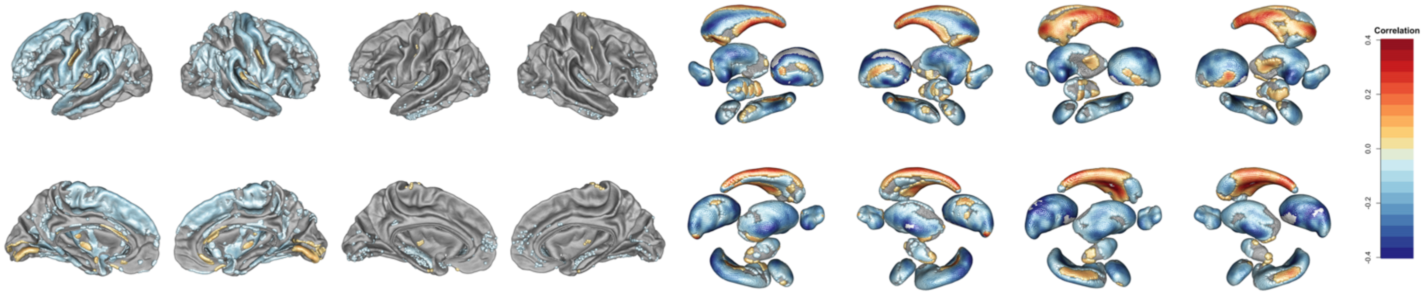

c)

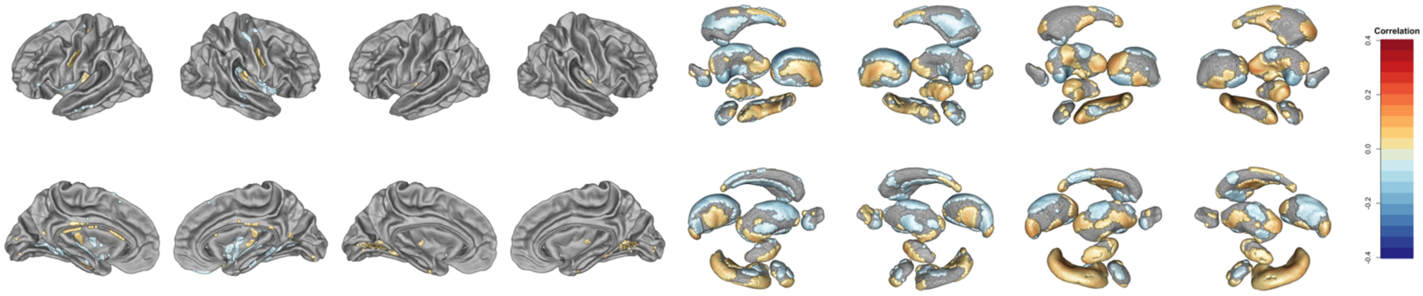

d)

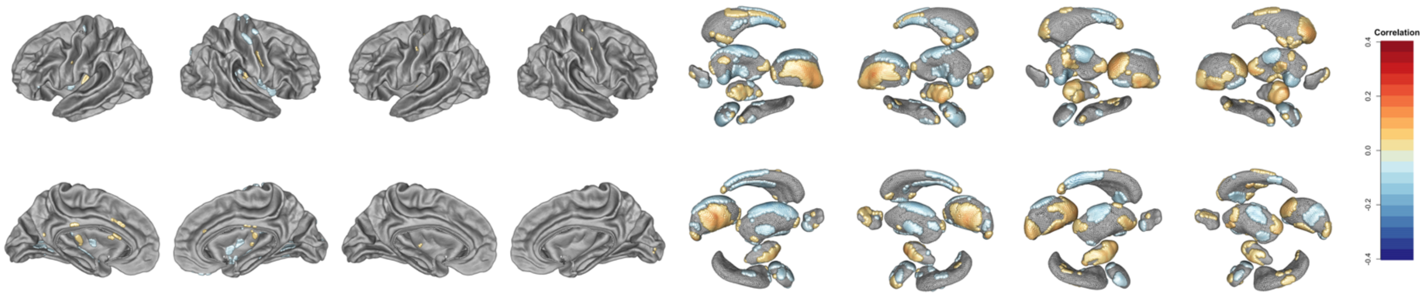

e)

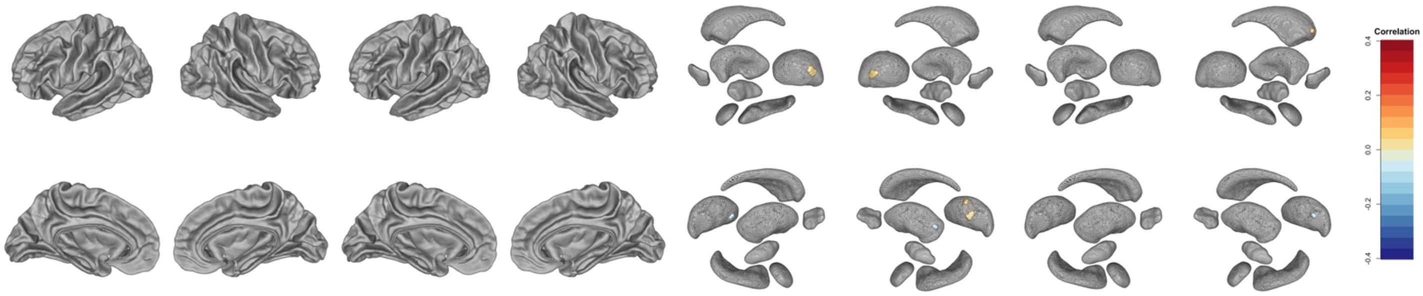

SFigure 12: Results of vertex-wise analysis for age at MRI using the different models

The brain plots present the significant vertices (in color) , for (from left to right) cortical thickness, cortical surface area, subcortical thickness and subcortical surface. The top and bottom rows shows the outside and inside view of the cortex and of the subcortical volumes. a) Results for GLM “no covariates”, b) GLM “sex, ICV”, c) GLM “5 global PCs”, d) GLM “10 global PCs, e) LMM “global BRM”.

a)

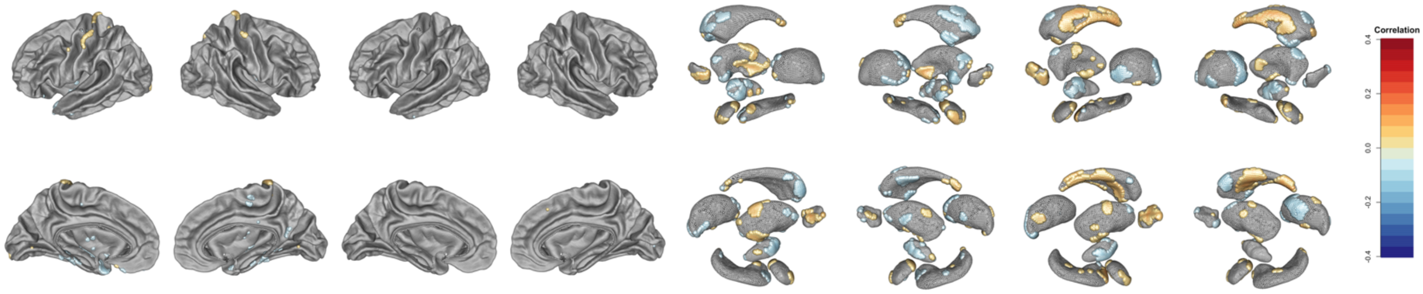

b)

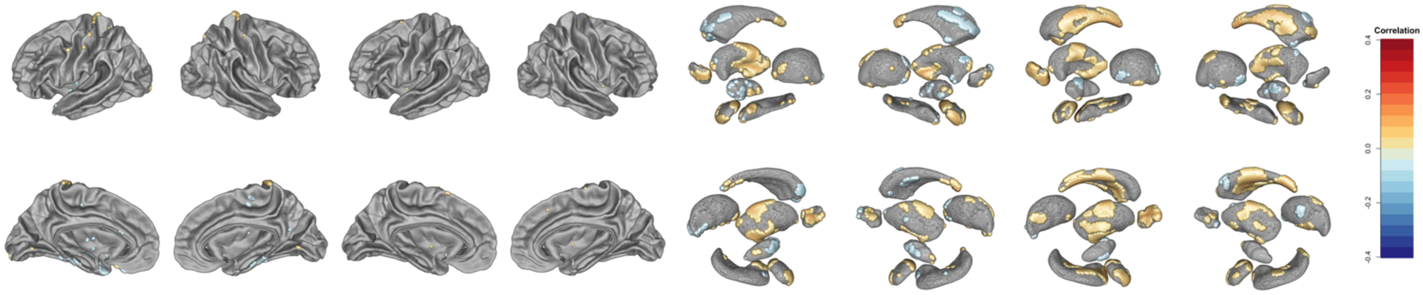

c)

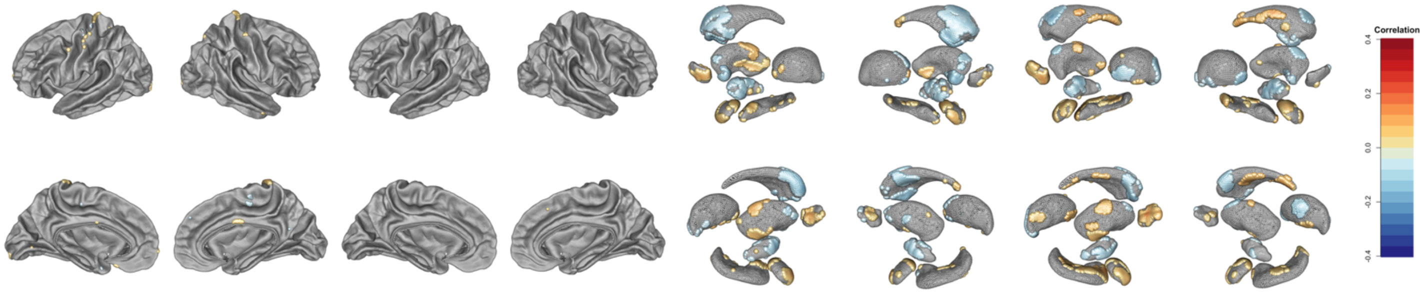

d)

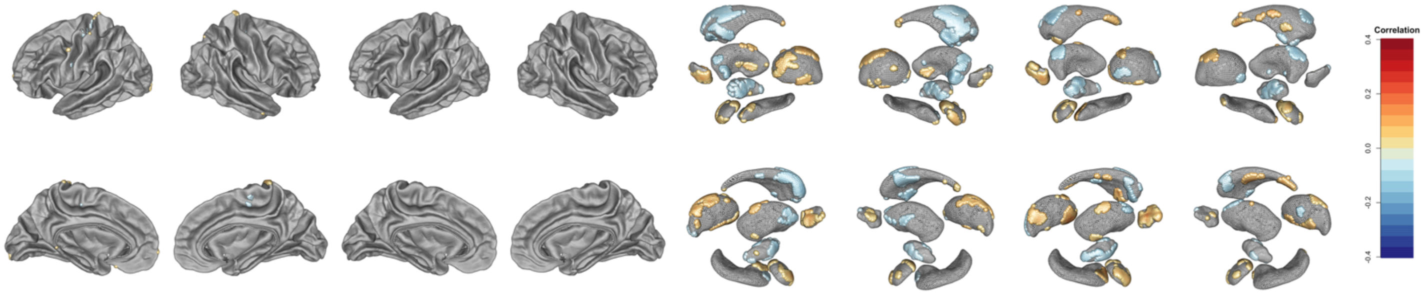

e)

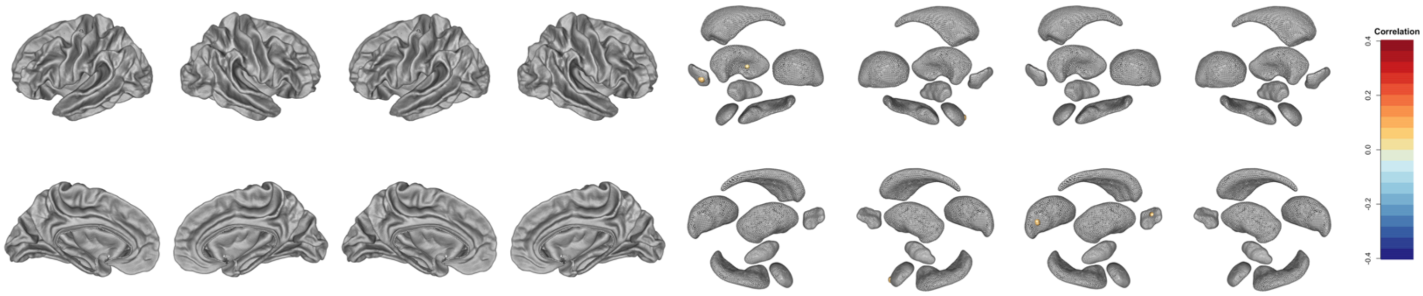

SFigure 13: Results of vertex-wise analysis for BMI using the different models

The brain plots present the significant vertices (in color) , for (from left to right) cortical thickness, cortical surface area, subcortical thickness and subcortical surface. The top and bottom rows shows the outside and inside view of the cortex and of the subcortical volumes. a) Results for GLM “no covariates”, b) GLM “sex, ICV”, c) GLM “5 global PCs”, d) GLM “10 global PCs, e) LMM “global BRM”.

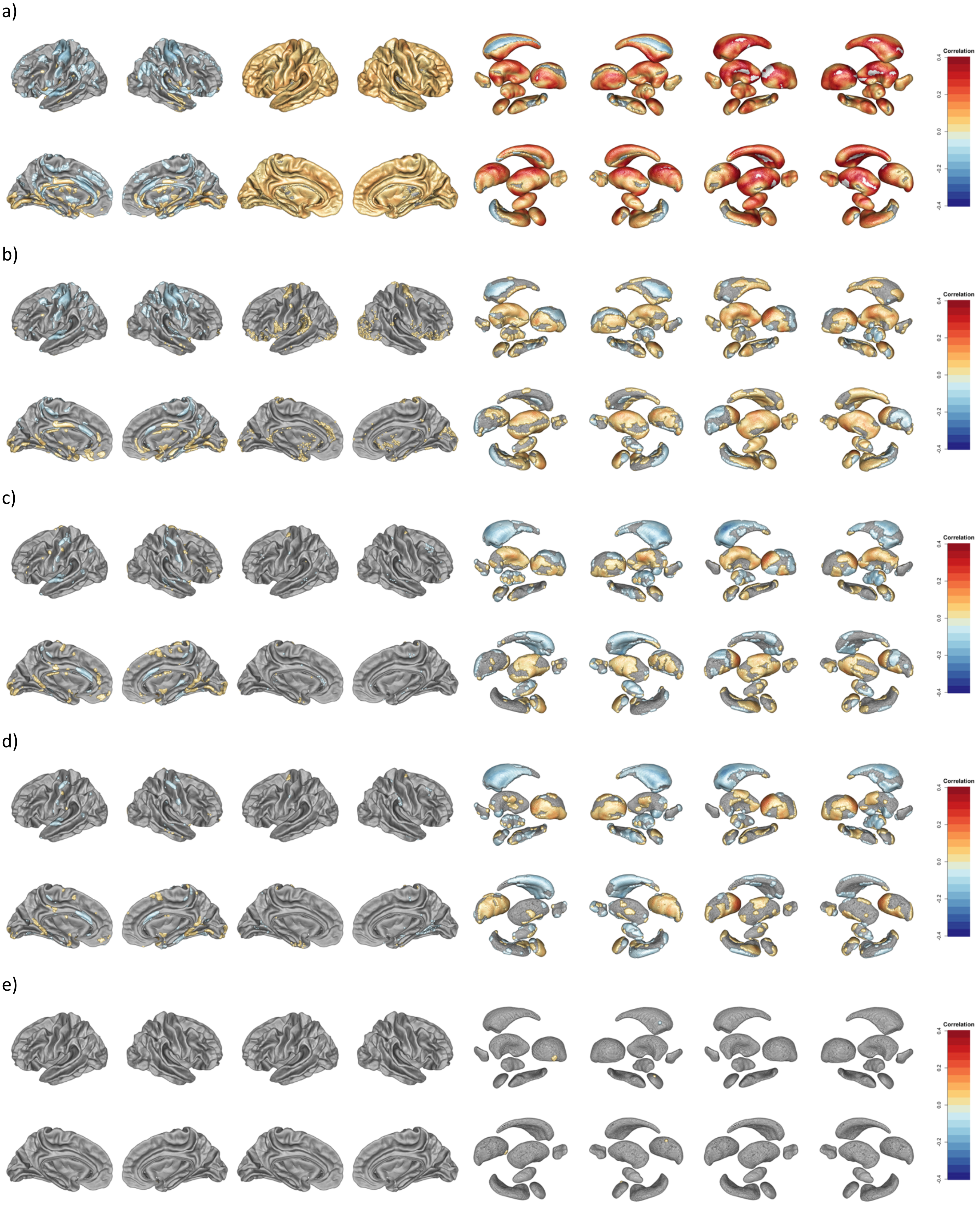

SFigure 14: Results of vertex-wise analysis for sex using the different models

The brain plots present the significant vertices (in color), for (from left to right) cortical thickness (left hemisphere, right hemisphere), cortical surface area, subcortical thickness and subcortical surface. The top and bottom rows shows the outside and inside view of the cortex and of the subcortical volumes. a) Results for GLM “no covariates”, b) GLM “sex, ICV”, c) GLM “5 global PCs”, d) GLM “10 global PCs, e) LMM “global BRM”.

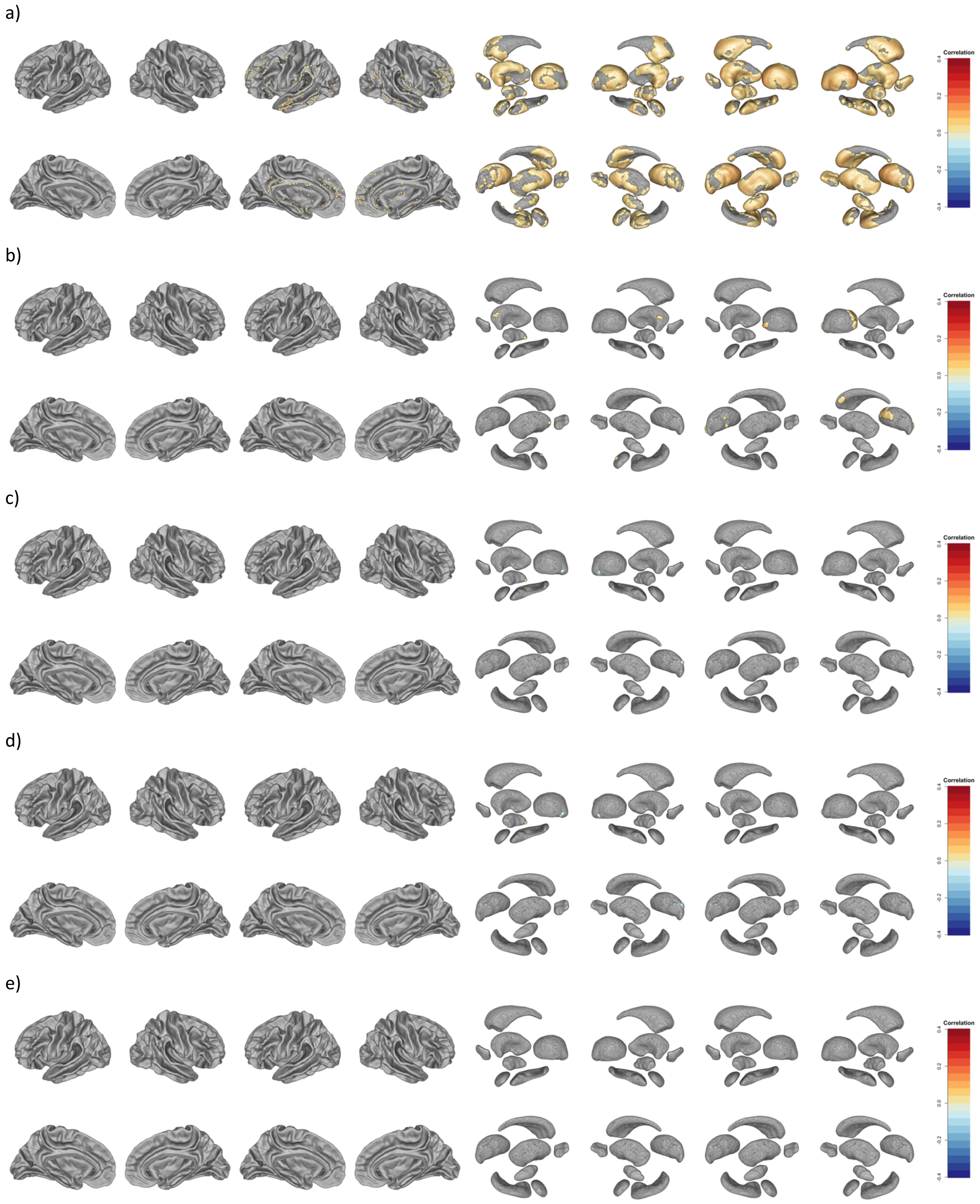

SFigure 15: Results of vertex-wise analysis for fluid IQ using the different models
The brain plots present the significant vertices (in color), for (from left to right) cortical thickness (left hemisphere, right hemisphere), cortical surface area, subcortical thickness and subcortical surface. The top and bottom rows shows the outside and inside view of the cortex and of the subcortical volumes. a) Results for GLM “no covariates”, b) GLM “sex, ICV”, c) GLM “5 global PCs”, d) GLM “10 global PCs, e) LMM “global BRM” (no significant association after multiple testing correction).

a)

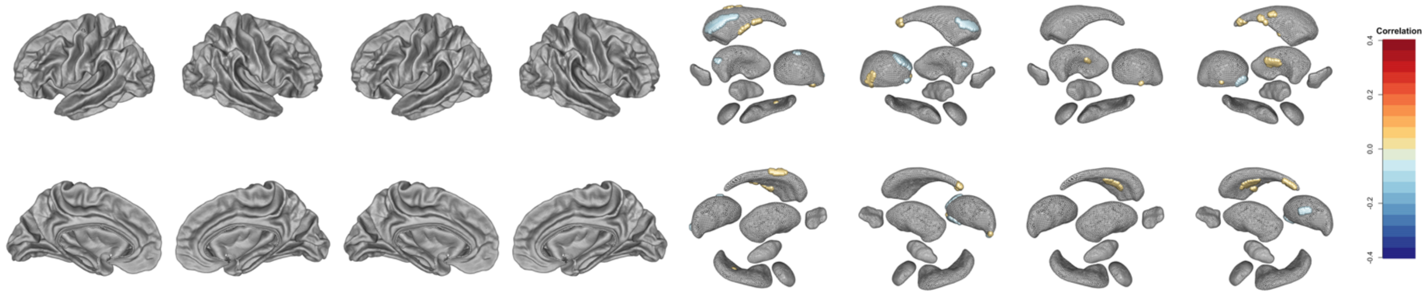

b)

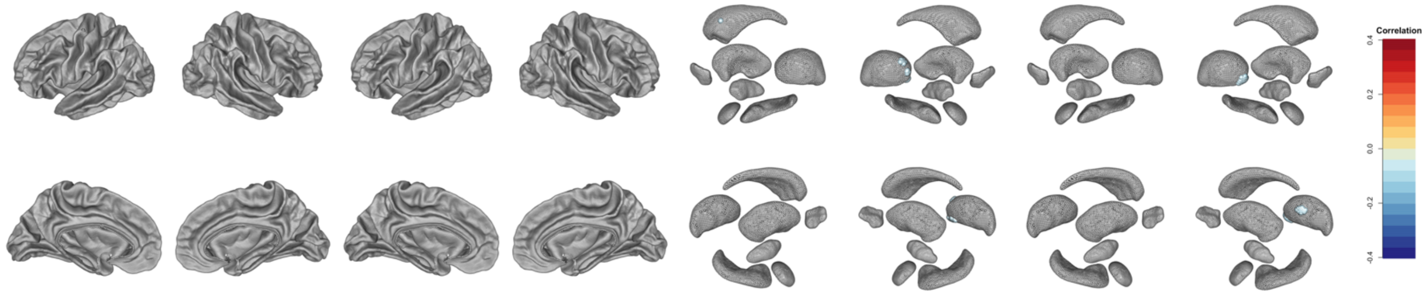

c)

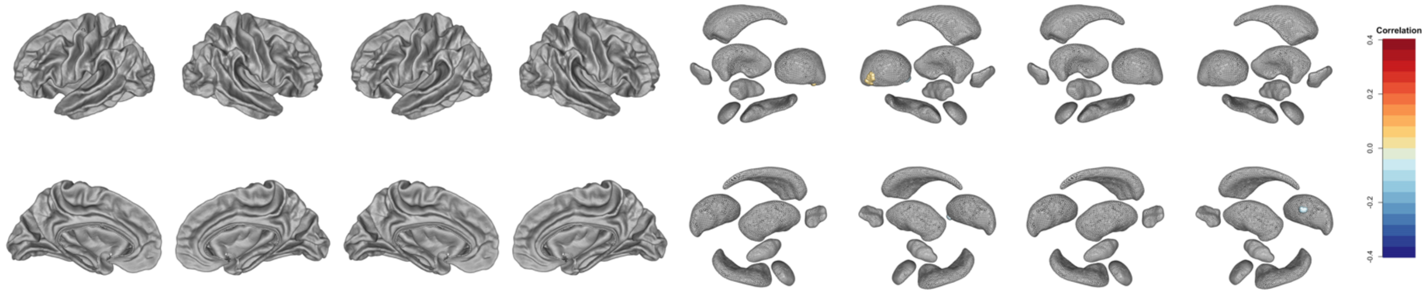

d)

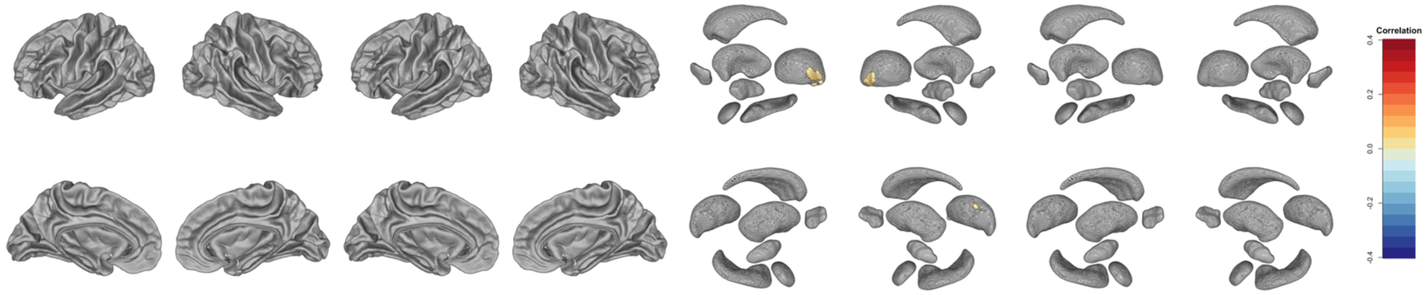

e)

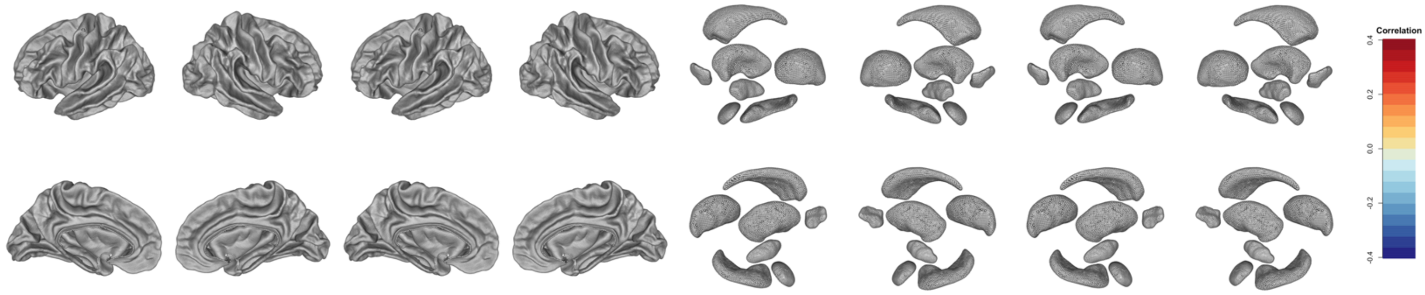

SFigure 16: Results of vertex-wise analysis for smoking status using the different models

The brain plots present the significant vertices (in color), for (from left to right) cortical thickness (left hemisphere, right hemisphere), cortical surface area, subcortical thickness and subcortical surface. The top and bottom rows shows the outside and inside view of the cortex and of the subcortical volumes. a) Results for GLM “no covariates”, b) GLM “sex, ICV”, c) GLM “5 global PCs”, d) GLM “10 global PCs, e) LMM “global BRM” (no significant association after multiple testing correction).

Metric	Num assoc. vertices	**No covariates**	**Age, Sex, ICV reg.**	**5 global PCs**	**10 global PCs**	**10 modality spe. PCs**	**LMM (global BRM)**	**LMM with covariates**	**LMM (multi. BRM)**
**Lambda (inflation factor)**	10	2.64 (1.58)	1.63 (0.64)	1.15 (0.07)	1.12 (0.05)	1.1 (0.04)	0.94 (0.02)	0.93 (0.02)	0.95 (0.02)
	100	6.45 (4.27)	3.01 (1.62)	1.46 (0.09)	1.37 (0.07)	1.31 (0.05)	0.91 (0.01)	0.9 (0.01)	0.91 (0.01)
	1000	4.42 (3.49)	2.53 (1.08)	1.48 (0.09)	1.37 (0.05)	1.32 (0.04)	0.96 (0.01)	0.96 (0.01)	0.97 (0.01)
**FPR (Nominal false positive rate)**	10	0.2 (0.11)	0.13 (0.06)	0.07 (0.01)	0.07 (0.01)	0.07 (0.01)	0.05 (<0.01)	0.04 (<0.01)	0.05 (<0.01)
	100	0.39 (0.16)	0.24 (0.1)	0.12 (0.01)	0.1 (0.01)	0.1 (0.01)	0.04 (<0.01)	0.04 (<0.01)	0.04 (<0.01)
	1000	0.3 (0.14)	0.21 (0.08)	0.12 (0.01)	0.1 (0.01)	0.09 (0.01)	0.05 (<0.01)	0.05 (<0.01)	0.05 (<0.01)
**True positive (TP) rate**	10	0.72 (0.12)	0.72 (0.12)	0.71 (0.13)	0.71 (0.13)	0.7 (0.13)	0.7 (0.13)	0.7 (0.13)	0.69 (0.13)
	100	0.45 (0.05)	0.43 (0.04)	0.42 (0.04)	0.41 (0.04)	0.41 (0.04)	0.38 (0.04)	0.38 (0.04)	0.37 (0.03)
	1000	0.05 (0.04)	0.03 (0.02)	0.02 (<0.01)	0.01 (<0.01)	0.01 (<0.01)	0.01 (<0.01)	0.01 (0)	0.01 (<0.01)
**Median size of TP cluster on cortical surface area**	10	11 (45)	7 (32)	3 (12)	2 (9)	1 (2)	1 (1)	1 (1)	1 (0)
	100	1 (0)	1 (0)	1 (0)	1 (0)	1 (0)	1 (0)	1 (0)	1 (0)
	1000	1 (0)	1 (0)	1 (0)	1 (0)	1 (0)	1 (0)	1 (0)	1 (0)
**Max. size** of TP cluster on cortical surface area	10	63 (183)	29 (79)	18 (49)	12 (37)	7 (22)	3 (8)	3 (8)	2 (3)
	100	163 (601)	7 (17)	3 (7)	3 (5)	2 (2)	1 (1)	1 (1)	1 (0)
	1000	205 (1283)	1 (2)	3 (19)	1 (0)	1 (0)	1 (0)	1 (0)	1 (0)
**Median size of TP cluster on cortical thickness**	10	152 (162)	149 (168)	124 (126)	112 (77)	107 (67)	79 (45)	79 (45)	77 (44)
	100	81 (63)	85 (172)	50 (12)	48 (13)	45 (13)	24 (8)	24 (8)	24 (7)
	1000	30 (22)	26 (16)	13 (7)	13 (8)	13 (7)	7 (5)	7 (5)	6 (5)
**Max. size** of TP cluster on cortical thickness	10	384 (502)	332 (304)	254 (178)	231 (142)	218 (130)	143 (65)	145 (64)	139 (62)
	100	1972 (8124)	1240 (5624)	226 (123)	214 (107)	196 (84)	86 (20)	86 (20)	85 (20)
	1000	225 (325)	196 (266)	73 (94)	50 (35)	44 (33)	11 (7)	11 (7)	10 (8)
**Median size of TP cluster sub-cortical surface**	10	795 (726)	447 (488)	236 (259)	164 (152)	104 (88)	93 (80)	88 (79)	49 (31)
	100	855 (769)	335 (434)	127 (132)	102 (99)	57 (39)	32 (22)	31 (20)	25 (17)
	1000	455 (551)	140 (205)	46 (57)	41 (49)	21 (17)	6 (2)	5 (4)	2 (NA)
**Max. size** of TP cluster sub-cortical surface	10	804 (732)	450 (486)	239 (257)	166 (151)	106 (87)	94 (79)	89 (78)	50 (31)
	100	1183 (889)	568 (608)	186 (210)	150 (131)	77 (57)	41 (30)	40 (28)	29 (18)
	1000	813 (846)	241 (370)	53 (63)	52 (55)	23 (18)	6 (2)	5 (4)	3 (NA)
**Median size of TP cluster on sub- cortical thickness**	10	747 (717)	384 (479)	206 (258)	193 (236)	82 (84)	64 (62)	61 (61)	32 (21)
	100	417 (495)	222 (249)	90 (101)	75 (81)	37 (36)	20 (19)	18 (17)	15 (11)
	1000	200 (220)	116 (270)	39 (44)	30 (30)	18 (30)	7 (10)	6 (9)	2 (2)
**Max. size** TP cluster on subcortical thickness	10	759 (713)	406 (481)	212 (257)	200 (234)	85 (83)	66 (63)	63 (61)	32 (21)
	100	668 (776)	385 (494)	148 (191)	116 (131)	51 (48)	26 (28)	24 (26)	17 (12)
	1000	554 (673)	188 (313)	50 (61)	39 (43)	21 (31)	7 (10)	6 (9)	2 (2)
**FWER**	10	1 (0)	1 (0)	1 (0)	1 (0)	1 (0)	1 (0)	1 (0)	1 (0)
	100	1 (0)	1 (0)	1 (0)	1 (0)	1 (0)	1 (0)	1 (0)	1 (0)
	1000	1 (0)	1 (0)	1 (0)	1 (0)	1 (0)	0.97 (0.17)	0.96 (0.2)	0.97 (0.17)
**Cluster FWER**	10	0.99 (0.01)	0.97 (0.02)	0.98 (0.01)	0.96 (0.02)	0.85 (0.04)	0.63 (0.05)	0.63 (0.05)	0.49 (0.05)
	100	1 (0)	1 (0)	1 (0)	0.99 (0.01)	0.97 (0.02)	0.72 (0.05)	0.7 (0.05)	0.72 (0.05)
	1000	1 (0)	1 (0)	1 (0)	0.99 (0.01)	1 (0)	0.7 (0.05)	0.67 (0.05)	0.58 (0.05)
**Statistical Power (cluster FWER<0.2)**	10	0.40 (0.01)	0.45 (0.01)	0.48 (0.01)	0.51 (0.01)	0.53 (0.01)	0.59 (0.01)	0.58 (0.01)	0.62 (0.013)
	100	0.03 (1.4E-3)	0.07 (1.9E-3)	0.13 (2.2E-3)	0.12 (2.2E-3)	0.16 (2.4E-3)	0.22 (2.7E-3)	0.22 (2.8E-3)	0.22 (2.7E-3)
	1000	3.6E-4 (1.5E-4)	4.1E-4 (8.2E-5)	6.5E-4 (9.4E-5)	6.9E-4 (8.6E-5)	6.9E-4 (8.6E-5)	2.4E-3 (1.4E-4)	2.4E-3 (1.4E-4)	2.0E-3 (1.3E-4)
**Cluster FDR**	10	0.75 (0.24)	0.64 (0.24)	0.48 (0.25)	0.41 (0.24)	0.32 (0.22)	0.16 (0.17)	0.16 (0.17)	0.12 (0.15)
	100	0.78 (0.18)	0.62 (0.17)	0.33 (0.16)	0.24 (0.13)	0.17 (0.1)	0.04 (0.03)	0.03 (0.03)	0.03 (0.03)
	1000	0.73 (0.13)	0.65 (0.13)	0.45 (0.16)	0.34 (0.13)	0.32 (0.13)	0.13 (0.11)	0.13 (0.12)	0.13 (0.15)
**Number of significant clusters**	10	96 (125)	33 (25)	20 (17)	15 (8)	12 (5)	9 (2)	9 (2)	13 (19)
	100	227 (204)	135 (86)	66 (21)	55 (12)	49 (8)	39 (4)	39 (4)	39 (7)
	1000	142 (155)	92 (77)	30 (17)	21 (7)	18 (5)	8 (3)	8 (3)	7 (3)
**Prediction (r) from top vertex per cluster**	10	0.29 (0.09)	0.35 (0.07)	0.38 (0.06)	0.4 (0.05)	0.42 (0.03)	0.43 (0.02)	0.43 (0.02)	0.43 (0.02)
	100	0.32 (0.09)	0.4 (0.08)	0.54 (0.08)	0.58 (0.06)	0.61 (0.04)	0.64 (0.02)	0.64 (0.01)	0.63 (0.02)
	1000	0.18 (0.04)	0.2 (0.05)	0.18 (0.05)	0.18 (0.05)	0.18 (0.04)	0.15 (0.04)	0.15 (0.04)	0.14 (0.04)
**Number of significant vertices**	10	4636 (7293)	1452 (1767)	747 (552)	616 (352)	492 (230)	325 (118)	320 (114)	261 (110)
	100	10596 (15205)	6840 (12859)	1967 (881)	1666 (595)	1309 (325)	565 (100)	557 (98)	510 (114)
	1000	5813 (13178)	2179 (2755)	345 (315)	209 (148)	150 (83)	23 (15)	23 (15)	20 (15)
	10	0.22 (0.06)	0.24 (0.05)	0.25 (0.05)	0.26 (0.06)	0.26 (0.05)	0.28 (0.06)	0.28 (0.06)	0.28 (0.06)
	100	0.23 (0.06)	0.27 (0.06)	0.34 (0.07)	0.36 (0.06)	0.38 (0.05)	0.4 (0.04)	0.4 (0.04)	0.4 (0.04)

**Prediction (r) from significant vertices**	1000	0.15 (0.05)	0.16 (0.06)	0.12 (0.05)	0.12 (0.05)	0.12 (0.04)	0.12 (0.04)	0.11 (0.03)	0.11 (0.03)
**AUC**	10	0.86 (0.06)	0.86 (0.06)	0.85 (0.07)	0.86 (0.07)	0.85 (0.07)	0.85 (0.06)	0.85 (0.07)	0.85 (0.07)
	100	0.71 (0.02)	0.71 (0.02)	0.71 (0.02)	0.71 (0.02)	0.7 (0.02)	0.69 (0.02)	0.69 (0.02)	0.68 (0.02)
	1000	0.52 (0.01)	0.51 (0.01)	0.51 (0)	0.51 (0)	0.51 (0)	0.5 (0)	0.5 (0)	0.5 (0)

STable 1: metrics of performance (SE) of mass-univariate models on simulated traits

The values are calculated over 100 simulated phenotypic traits.

STable 2: Summary of mass-univariate vertex-wise analyses for the UKB phenotypes considered

		**BMI**	**Fluid Intelligence**	**Age**	**Sex**	**Smoking status**
	Adj. R^2 with age, sex & ICV	0.011	0.030	0.012	0.31	0.0086
	Adj. R^2 with first 10 PCs	0.033	0.032	0.41	0.43	0.0092
**Uncorrected GLM**	*N assoc. vertices*<br>*N assoc. clusters*<br>*Max cluster size*<br>*Prediction (UKB)*<br>*Prediction (OASIS3)*	10,947<br>232<br>862<br>0.41 [0.38,0.45]<br>0.26 [0.19,0.33]	24,776<br>640<br>2,030<br>0.14 [0.08,0.21]<br>-0.05 [-0.16,0.06]	136,278<br>970<br>22,358<br>0.70 [0.68,0.73]<br>0.75 [0.7,0.8]	355,130<br>714<br>130,651<br>0.65 [0.61,0.68]<br>0.42 [0.34,0.5]	707<br>34<br>116<br>0.12 [0.08,0.16]<br>-0.03 [-0.13,0.07]
**Age, sex, ICV GLM**	*N assoc. vertices*<br>*N assoc. clusters*<br>*Max cluster size*<br>*Prediction (UKB)*<br>*Prediction (OASIS3)*	10,240<br>237<br>494<br>0.37 [0.34,0.4]<br>0.21 [0.15,0.28]	195<br>15<br>112<br>0.12 [0.07,0.16]<br>0.02 [-0.06,0.1]	129,700<br>1269<br>19,450<br>0.74 [0.71,0.76]<br>0.76 [0.71,0.81]	321,154<br>1154<br>2,955<br>0.74 [0.71,0.78]<br>0.59 [0.51,0.66]	88<br>6<br>37<br>0.08 [0.04,0.12]<br>-0.01 [-0.09,0.07]
**5 global PCs GLM**	*N assoc. vertices*<br>*N assoc. clusters*<br>*Max cluster size*<br>*Prediction (UKB)*<br>*Prediction (OASIS3)*	10,407<br>201<br>680<br>0.35 [0.32,0.38]<br>0.22 [0.15,0.29]	24<br>5<br>10<br>0.06 [0.03,0.1]<br>-0.01 [-0.07,0.06]	28,166<br>385<br>1,875<br>0.37 [0.34,0.4]<br>0.19 [0.13,0.24]	31,640<br>499<br>1,618<br>0.5 [0.48,0.52]<br>0.38 [0.34,0.42]	39<br>4<br>25<br>0.04 [0,0.07]<br>-0.04 [-0.11,0.03]
**10 global PCs GLM**	*N assoc. vertices*<br>*N assoc. clusters*<br>*Max cluster size*<br>*Prediction (UKB)*<br>*Prediction (OASIS3)*	8,548<br>174<br>518<br>0.35 [0.31,0.38]<br>0.16 [0.09,0.23]	30<br>5<br>9<br>0.06 [0.03,0.1]<br>-0.01 [-0.07,0.06]	16,746<br>297<br>894<br>0.32 [0.29,0.34]<br>0.14 [0.08,0.2]	26,894<br>492<br>1782<br>0.44 [0.42,0.46]<br>0.34 [0.29,0.39]	91<br>6<br>43<br>0.01 [-0.02,0.04]<br>-0.07 [-0.14,0]
**10 moda. Spe. PCs GLM**	*N assoc. vertices*<br>*N assoc. clusters*<br>*Max cluster size*<br>*Prediction (UKB)*<br>*Prediction (OASIS3)*	4,227<br>161<br>346<br>0.33 [0.3,0.36]<br>0.15 [0.08,0.21]	75<br>4<br>37<br>0.07 [0.03,0.1]<br>0 [-0.07,0.06]	13,367<br>404<br>616<br>0.65 [0.63,0.68]<br>0.7 [0.65,0.75]	18,869<br>515<br>1437<br>0.54 [0.52,0.56]<br>0.45 [0.4,0.49]	17<br>3<br>6<br>0.04 [0.01,0.07]<br>-0.04 [-0.1,0.02]
**Single random effect LMM**	*N assoc. vertices*<br>*N assoc. clusters*<br>*Max cluster size*<br>*Morphometricity (SE)*<br>*Prediction (UKB)*<br>*Prediction (OASIS3)*	11<br>5<br>5<br>0.58 [0.54,0.64]<br>0.16 [0.13,0.19]<br>0.09 [0.01,0.17]	0<br>0<br>-Inf<br>0.16 [0.12,0.21]<br>NA [NA,NA]<br>NA [NA,NA]	47<br>8<br>15<br>0.91 [0.87,0.95]<br>0.45 [0.42,0.48]<br>0.35 [0.29,0.42]	27<br>6<br>11<br>0.99 [0.96,1.04]<br>0.28 [0.25,0.31]<br>0.22 [0.17,0.28]	0<br>0<br>-Inf<br>0.15 [0.10,0.19]<br>NA [NA,NA]<br>NA [NA,NA]
**LMM with covariates**	*N assoc. vertices*	3	0	40	20	0
	N assoc. clusters	1	0	6	3	0
	Max cluster size	3	-Inf	12	10	-Inf
	Morphometricity (SE)	0.57 [0.52,0.62]	0.15 [0.10,0.19]	0.90 [0.85,0.93]	0.99 [0.96, 1.04]	0.13 [0.092,0.17]
	Prediction (UKB)	0.07 [0.04,0.1]	NA [NA,NA]	0.31 [0.28,0.34]	0.2 [0.17,0.23]	NA [NA,NA]
	Prediction (OASIS3)	0.1 [0.03,0.18]	NA [NA,NA]	0.15 [0.09,0.22]	0.16 [0.11,0.21]	NA [NA,NA]
**Multiple random effect LMM**	*N assoc. vertices*<br>*N assoc. clusters*<br>*Max cluster size*<br>*Morphometricity (SE)*<br>*Prediction (UKB)*<br>*Prediction (OASIS3)*	0<br>-Inf<br>NA<br>NA<br>NA<br>NA	0<br>-Inf<br>NA<br>NA<br>NA<br>NA	0<br>-Inf<br>NA<br>NA<br>NA<br>NA	1<br>9<br>9<br>1.07 [1.02, 1.11]<br>0.14 [0.11,0.16]<br>0.15 [0.1,0.19]	0<br>-Inf<br>NA<br>NA<br>NA<br>NA

In the "age, sex and ICV adjusted" GLM, we dropped the corresponding covariate when studying age and sex. All adjusted R^2 are significant (pvalue<1e-16) considering the large sample size. Significance corresponded to a Bonferroni significance threshold of 1.5e-8, which accounts for the number of vertices and traits analyses.

STable 3: top vertices in each significant cluster from LMM single random effect models

	Top vertex					Discovery					Replication UKB		Replication OASIS3	
		**hemi**	**modality**	**region**	**X;Y;Z coordinates**	**beta**	**se**	**pvalue**	**r**	**Cluster size**	**beta**	**pvalue**	**beta**	**pvalue**
BMI	LogJacs_10_738	Left	Subcort. surface	Thalamus-Proper	148.5 ; 110.9 ; 105.1	0.363	0.063	9.7E-09	0.082	2	0.029	7.3e-01	-0.23	0.25
	LogJacs_26_484	Left	Subcort. surface	Accumbens-area	136.3 ; 109.1 ; 144.7	0.408	0.072	1.5E-8	0.092	1	0.139	1.3e-01	0.098	0.63
	thick_12_879	Left	Subcort. thickness	Putamen	152.4 ; 111.8 ; 125.4	0.316	0.055	1.2E-8	0.072	1	0.22	3.6e-03	0.072	0.69
	thick_26_291	Left	Subcort. thickness	Accumbens-area	132.3 ; 114 ; 141.6	0.38	0.063	1.4E-09	0.086	5	0.359	3.2e-05 *	0.18	0.41
	thick_54_1331	Right	Subcort. thickness	Amygdala	106.2 ; 131.8 ; 141.1	0.447	0.075	2.7E-09	0.101	2	0.229	1.8e-02	0.20	0.42
Age	LogJacs_49_1110	Right	Subcort. surface	Thalamus-Proper	108.9 ; 105.4 ; 108.6	-0.552	0.097	1.3E-8	-0.074	2	-0.388	2.4e-03	-1.24	4.8E-06 *
	LogJacs_50_2347	Right	Subcort. surface	Caudate	119.8 ; 100.6 ; 151.3	0.64	0.11	1.3E-8	0.086	1	0.388	1.2e-02	1.15	1.2E-4 *
	thick_10_2231	Left	Subcort. thickness	Thalamus-Proper	139.5 ; 109.8 ; 125.6	-0.623	0.094	3.7E-11	-0.084	5	-0.658	5.6e-07 *	-0.56	1.0E-02
	thick_10_732	Left	Subcort. thickness	Thalamus-Proper	130.3 ; 107.4 ; 104.7	0.581	0.091	1.9E-10	0.078	15	0.793	2.9e-10 *	0.091	7.0E-1
	thick_49_954	Right	Subcort. thickness	Thalamus-Proper	126 ; 107 ; 107.3	0.618	0.088	1.8E-12	0.083	11	0.577	3.4e-07 *	0.75	1.7E-3
	thick_49_1337	Right	Subcort. thickness	Thalamus-Proper	110.2 ; 104.4 ; 111.3	0.536	0.081	3.2E-11	0.072	9	0.517	2.5e-04 *	0.47	1.0E-1
	thick_49_1652	Right	Subcort. thickness	Thalamus-Proper	113.4 ; 97.9 ; 115.5	0.625	0.10	1.7E-09	0.084	2	0.76	3.2e-10 *	0.78	8.8E-4 *
	thick_51_243	Right	Subcort. thickness	Putamen	98.6 ; 109.8 ; 117.3	-0.43	0.070	9.1E-10	-0.058	2	-0.348	5.6e-04 *	-0.68	1.8E-03
Sex	rht_46930	Right	Cort. thickness	Lateral orbitofrontal	-14 ; 11.2 ; -14.8	0.031	0.0043	1.3E-12	0.061	10	0.022	2.7e-04 *	0.045	2.6E-04 *
	thick_10_1216	Left	Subcort. thickness	Thalamus-Proper	128.5 ; 113.5 ; 109.3	0.038	0.0058	1.2E-10	0.076	11	0.034	3.5e-05 *	0.046	1.7E-3
	thick_10_2415	Left	Subcort. thickness	Thalamus-Proper	136.1 ; 111.9 ; 127.4	0.029	0.0046	4.3E-10	0.058	3	0.028	2.3e-05 *	0.016	2.1E-01
	thick_49_1483	Right	Subcort. thickness	Thalamus-Proper	113.3 ; 97.8 ; 113.2	0.037	0.0064	9.2E-09	0.074	1	0.027	2.3e-03	0.074	1.8E-05 *
	thick_50_1785	Right	Subcort. thickness	Right-Caudate	123.2 ; 98.6 ; 141.6	-0.034	0.0059	6.7E-09	-0.068	1	-0.031	1.3e-04 *	-0.030	4.4E-02
	thick_54_597	Right	Subcort. thickness	Amygdala	111.4 ; 121.1 ; 131.8	0.026	0.0046	1.2E-8	0.053	1	0.031	1.4e-06 *	0.064	1.6E-06 *

**: significant in the replication sample after multiple testing correction (p<0.05/85=5.8e-4).*

STable 4: review of mass-univariate analyses of Age

Article	DOI	Year	Phenotype	N	population	Matching / covariates	Vertex modalities	Smoothing / mesh	Multiple testing	Significant regions
Medic et al.,	10.1038/ijo.2016.42	2016	Age	202	Healthy adults	Age, sex, scanner, BMI, hemisphere, global thickness, area)	Cortical thickness, area	15mm	Cluster level using Monte Carlo simulations	11 regions in cortical thickness
Harrison et al.,	10.1016/j.neurobiolaging.2018.03.024	2018	Successful ageing (cognition)	129	Older adults (70+)	sex	Cortical thickness	NS	No correction	Impossible to conclude
Dotson et al.,	10.3389/fnagi.2015.00250	2016	Age	46	Middle aged adults (51-81 years	Sex, education, ICV/CT	Cortical thickness, surface	10mm	FDR	9 cortical regions surface area
Ducharme et al.,	10.1016/j.neuroimage.2015.10.010	2016	Age	384	HC 4-22 years old, longitudinal (753 scans)	Sex, scanner (TBV)	Cortical thickness	20mm	RFT peak and clusters	Linear model good approx. for most vertices
Li et al.,	10.1093/cercor/bhs413	2013	age	73	Imaged at birth and 2 years, longitudinal	NA	Asymetry in sulcal depth, surface area, curvature. Left right SA ratio	NA	sufstat	Regional asymetries found for several modalities
Hogstrom	10.1093/cercor/bhs231	2012	Age	322	Healthy adults (20-85)	Sex, Total WM volume	Cortical thickness, surface, gyrification	30mm	FDR Genovese et al., 2002	Surface area showed strong age-related decreases, particularly pronounced in dorsomedial prefrontal, lateral temporal, and fusiform cortices, independently of total white matter volume.
Hugues et al.,	10.1016/j.neuroimage.2012.07.043	2012	Age	86	Healthy subjects (20-74)	none	Thalamus shape/expansion	NA	FDR (non-specified)	Most of thalamus vertices significant
Sowell et al.,	10.1523/JNEUROSCI.1798-04.2004	2004	Age	45	Imaged twice, age 5-11. T-test between t1 and t2 (no mixed models).	none	Cortical thickness (Eikonal Fire Equation, not FreeSurfer) 65K vertices p.h.	15mm	Permutation to estimate minimal area significant	10 significant regions
Muftuler et al.,	10.1016/j.brainres.2011.05.018	2011	Age	126	Normally developing children age 6-10	none	Cortical thickness (FS)	Unclear	FDR (unspecified)	Many cortical regions significant
Reid	10.1002/hbm.20994	2010	Age	503	nondemented elderly individuals (50–85 years) with a history ofsymptomatic cerebral small vessel disease (SVD	sex	Cortical thickness (CIVET) 40,962 vertices	NA	RFT	Most of the cortical sheet showed significant decrease with Age, with the greatest effects apparent in the ventrolateral prefrontal cortex(BA45, BA46, and BA47), the primary and secondary audi-tory cortices (BA41, BA42), Wernicke’s area (BA22), medialtemporal lobe (BA36, BA28, excluding the hippocampal formation and amygdala), and the primary visual cortex.
Salat et al.,	10.1093/cercor/bhh032	2004	Age	106	non-demented participants ranging in age from 18 to 93 years Imaged several times with T1 averaged	sex	Cortical thickness (FS)	22mm	none	Some regions likely significant after bonferroni correction.
Gogtay	10.1073pnas.0402680101	2004	Age	13	Healthy age 4-21. Up to 4 scans per subject (52 images)	none	Cortical GM density	15mm	None	Results not interpretable

							(Thompson et al., 2000)			
Gogtay	10.1002/hipo.20193	2006	Age	31	Healthy age 4-21. Up to 4 scans per subject (100 images)	Sex, TBV	Hippocampus thickness, manual tracing, 30,000 measurements	NA	none	Results not interpretable
Van Soelen	10.1016/j.neuroimage.2011.11.044	2012	Age	113	Healthy twins 9–13 Imaged twice with 3 years interval	Sex, handedness, scan interval	Cortical thickness (CLASP) 40 962 vertices per hemisphere	20mm	FDR (Genovese et al.,)	Widespread significant regions
Tamnes	10.1523/JNEUROSCI.3302-16.2017	2017	Age	85	Healthy adolescents, up to 2 scans per individual, 170 images total. 4 samples	Sex, scanning interval	Cortical thickness, volume, surface (FS)	15mm	Monte carlo simulations, clusters p<0.05	Widespread significant regions

STable 5: Review of mass-univariate analyses of Sex

Article	DOI	Year	Phenotype	N	population	Matching / covariates	Vertex/voxel modalities	Smoothing / mesh	Multiple testing	Significant regions
Lotze et al.,	10.1038/s41598-018-38239-2	2018	Sex	2,838	Adults age 21-90	TBV, IQR, age, years of education, nicotine intake, alcohol consumption, and body mass index (BMI)	VBM	8mm	FWER (not specified), cluster size >10 voxels	25 significant regions
Chen et al.,	10.1016/j.neuroimage.2007.03.063	2007	Sex	411	Adults, 44-48 years	age, years of education, handedness, and total intracranial volume	Cortical volume (VBM)	12mm	FWER (not specified), threshold p<0.001	15 significant regions
Ruigrok et al.,	10.1016/j.neubiorev.2013.12.004	2014	Sex	<2186	Meta-analysis all ages. Incl. Chen et al., 2007	Depending on study	Cortical volume, density (VBM)	Depends on study	FDR	22 regions associated. Meta-analysis relies on foci, not on full map of summary statistics.
Jiang et al.,	10.1371/journal.pone.0073932	2013	Sex	266	Inflamattory Bowel Disease (90). 176 HC	Age, Total grey matter volume	Cortical thickness	8mm	FDR (RFT)	4 cortical regions
Li et al.,	10.1093/cercor/bhs413	2013	sex	73	Imaged at birth and 2 years, longitudinal	age	Asymetry in sulcal depth, surface area, curvature		sufstat	
Boulos et al.,	10.1371/journal. pone.0152983	2016	Sex	87	Right handed females (14-19 years) and males (14-18, See Chumachenko et al.,)	Age, IQ	Cortical thickness	10mm	p<0.005, monte carlo simulations: cluster >250 mm2	No significant findings
Richie et al,.	10.1093/cercor/bhy109	2018	Sex	5216	UK Biobank	Age, ethnicity	Cortical thickness, surface area, volume (FS) Rs-fMRI WM microstructure	20mm	None (post hoc analysis)	Results compared to ROI based results.<br>Tred: large SQ and VOL in males, larger thickness in females
Luders et al	10.1002/hbm.20187	2005	Sex	60	Healthy young adults, right handed, matched for age	2 processing, one accounting for global head size in realignment	Cortical thickness (Eikonal fire equations)	15mm	permutation	Increased CT in females, widespread. Esp. when accounting for head size
Lv et al.,	10.1016/j.neuroimage.2010.05.020	2010	Sex	184	Healthy adults (18-70)	Age, head size (GM+WM+CSF)	Cortical thickness (no FS) 40,962 p.h.	20mm	FDR (Genovese et al.,)	cortical thickening in females appeared extensively in the frontal, parietal and occipital lobes, including the superior frontal gyrus, precentral gyrus, and postcentralgyrus in both hemispheres, and the superior parietal lobule, cuneus, and frontal pole in left hemispheres. The male cortex was significantly thicker than that of the female only in some small regions of the temporal lobes.
Sowell et al.,	10.1093/cercor/bhl066	2007	Sex	176	Healthy 7-87 years	Age, age2, (height)	Cortical thickness		Permutations (within large	Results of ROI permutation analyses (shown in Table 2) confirm the significance of sex differences in

							(manual and automated processing) Eikonal fire equation 65536 vertices p.h.		cortical regions) to estimate cluster size	cortical thickness in right lateral parietal (P = 0.048), right lateral temporal (P= 0.024), and left medial occipital (P = 0.017) regions.
				36	Follow up in male female sample matched for TBV					Female thicker. Right: Lateral ventral frontal<br>Lateral occipital<br>Lateral parietal<br>Lateral temporal
Van Velsen	10.1016/j.neulet.2013.06.063	2013	Sex	1022	Non-demented elderly: age ~68	ICV, education (in men and women separately)	Cortical thickness (FS)	NA	none	Results not presented/discussed. ROI focus
Reid	10.1002/hbm.20994	2010	Sex	503	nondemented elderly individuals (50–85 years) with a history ofsymptomatic cerebral small vessel disease (SVD	age	Cortical thickness (CIVET) 40,962 vertices	NA	RFT	Vertex-wise analyses highlightsome regions where a moderate Sex effect was apparent.AfterP-value correction, these effects were not significant;

STable 6: Review of mass-univariate analyses of smoking (and related traits)

Article	DOI	Year	Phenotype	N	population	Matching / covariates	Vertex modalities	Smoothing / mesh	Multiple testing	Significant regions
Jorgensen et al.,	10.1503/jpn.140163	2015	Smoking	743	237 healthy controls and 506 psychiatric cases	Age, sex, diagnosis	Cortical thickness	20mm	FDR (no reference)	1 cluster significant in patient sample
			Smoking amount							No significant result
Cox et al.,	10.1093/eurheartj/ehz100	2019	Vascular risk factors (BMI, smoking)	7928	UKB adults	age, sex, ethnicity, head size (for volumetric data), and head positioning confounds	Cortical volume	20mm	FDR (Benjamini Hochberg)	Several large cortical regions (lateral and medial temporal lobes)
Chye et al.,	10.1111/adb.12830	2019	Substance dependence (incl. nicotine)	3905	Multiple substance dependence (ENIGMA)	Site, sex, age, and ICV	Subcortical thickness and area	none	FDR (Landers et al.,)	Several subcortical volumes associated
Boulos et al.,	10.1371/journal.pone.0152983	2016	Substance abuse (10 substances, incl. tobacco)	43	Right handed females (14-19 years)	Age, IQ	Cortical thickness	10mm	p<0.005, monte carlo simulations: cluster >250 mm2	pregenual rostral anterior cingulate cortex extending to the medial orbitofrontal cortex
Chumachenko	10.3109/00952990.2015.1058389	2015	Substance use (and conduct problems)	44	Males 14-18	Age, IQ, total cortical thickness	Cortical thickness	NA	FWER – cluster leve; - Monte Carlo simulation (10,000 iterations) with a cluster-forming threshold vertex-level p-value of 0.005 (55)	Left posterior cingulate/precuneus

STable 7: Review of mass-univariate analyses of BMI (or related traits)

Article	DOI	Year	Phenotype	N	population	Matching / covariates	Vertex modalities	Smoothing / mesh	Multiple testing	Significant regions
Medic et al.,	10.1038/ijo.2016.42	2016	BMI	202	Healthy adults	Age, sex, scanner, hemisphere, global thickness, area, (smoking status)	Cortical thickness, area	15mm	Cluster level using Monte Carlo simulations	2 clusters in cortical thickness
Sharkey et al.,	10.3389/fnins.2015.00024	2015	BMI	378 (716 scans)	Healthy children (<18), longitudinal MRIs	Age, sex, scanner	Cortical thickness (CIVET)	20mm	FDR correction (Non-specified, surfstat?)	No significant association
Bernardes et al.,	10.1007/s11011-018-0223-5	2018	BMI / obesity	31 lean normoglycemic controls<br>44 obese	28 Obese with T2Diabetes<br>Age 40-70	Age, sex, hypertention and ICV	Cortical thickness, area, volume	10mm	Monte-carlo simulations, Pthreshold <0.01	1 cortical thickness cluster
Veit et al.,	10.1016/j.nicl.2014.09.013	2014	BMI	72	Healthy subjects age 19-50	Age, sex, total surface area, education	Cortical thickness	10mm	Monte carlo threshold estimation, after cutoff P<0.05	3 thickness clusters
Varma et al.,	10.1002/hipo.22586	2016	Physical activity	90	Adults > 6o years	intracranial volume (ICV), age, years of education, body mass index (BMI), cardiovascular disease burden (CVD), and global cognitive function	Subcortical shape	NA	FWER and FDR	Some significant regions (hippocampus)
Cox et al.,	10.1093/eurheartj/ehz100	2019	Vascular risk factors (BMI, smoking)	7928	UKB adults	age, sex, ethnicity, head size (for volumetric data), and head positioning confounds	Cortical volume	20mm	FDR (Benjamini Hochberg)	Several large cortical regions (lateral and medial temporal lobes)
Leritz et al.,	10.1016/j.neuroimage.2010.10.050.	2011	Cerebrovascular health (PCs derived from BMI)	115	Healthy controls, age 43-83	age	Cortical thickness	20mm	Clustering, after P<0.05	Comparison with other results impossible

STable 8: Review of mass-univariate analyses of IQ/cognition

Article	DOI	Year	Phenotype	N	population	Matching / covariates	Vertex modalities	Smoothing / mesh	Multiple testing	Significant regions
Harrison et al.,	10.1016/j.neurobiolaging.2018.03.024	2018	Successful ageing (cognition)	129	Older adults (70+)	sex	Cortical thickness	NS	No correction	Impossible to conclude
Abe et al.,	10.1111/acps.12922	2018	Executive functionning	160	HC, Type I and II bipolar	Sex (no age) + lot more in sensitivity analyses	Cortical thickness	10mm	Monte carlo, cluster wise. Threshold p<0.05	Several regions, some found across disease groups
Navas-Sanchez	10.1002/hbm.23143	2016	Math gifted	62	Spanish adolescents – IQ matched	age, gender, and IQ	Cortical thickness, area, volume	15mm	Cluster wise probability method (FDR) Hagler et al., 2006	Surface and thickness associated regions
Burgaleta	10.1002/hbm.22305	2014	Fluid IQ (and other IQ dimensions)	104	Psychology undergraduates (age ~19)	Age, sex (brain size in processing)	Subcortical shape/deformation	NA	FDR	right hemisphere only, for the accumbens, caudate, and putamen.
Burgaleta	10.1016/j.neuroimage.2013.09.038.	2014	IQ change	188	Healthy adolescents 6-20	Sex, scanner, time to repeat IQ	Cortical thickness, area	20mm (thick) 40mm (area)	Sufstat 5,000 permutations (Nichols and Holmes, 2002)	3 SA regions
Walhovd	10.1016/j.neuroimage.2006.01.011	2006	Memory recall (5 mins, 30 mins, 83 days)	71	Healthy adults 40+	gender, age, IQ, and intracranial volume, (hippo volume)	Cortical thickness	12.6mm	Uncorrected	Un-interpretable
Voineskos	10.1002/hbm.22825	2015	Cognition (verbal episodic memory, visuospatial episodic memory, and working memory)	137	Healthy adults 18-86	Age, sex, education, APOE e4 status	Hippocampus shape (normalised fro TBV)	NA	10% FDR (and 5%) (Genovese et al.,)	No significant associations at FDR <5%
Winjen	10.1007/s00330-019-06437-9	2019	EDSS and cognition domains	34	relapsing-remitting multiple sclerosis	Age, ICV	T1, T2, T2*, PD in grey matter masks	10mm	Monte carlo, p<0.05	T2 associations (no multiple correction for number of phenotypes studied)

Bobholz	10.1007/s11682-018-0005-z	2019	Cognition domains focus: psychomotor speed (digit symobol)	135	81 idiopathic epilepsies<br>54 healthy controls	age, gender, and IQ, (epilepsy)	CV, CT, SA, and LGI (local gyrification index)	15mm	Use of Qdec's Monte Carlo simulation allowed for corrections of multiple comparisons, with the cluster forming threshold set to $p < 0.05$	LGI associations: left postcentral gyrus, left lateral occipital gyrus, and right caudal middle frontal gyrus
Brathen	10.1002/hbm.24287	2018	Episodic memory plasticity (improvment)	126	HC, did a 10weeks memory course<br>2 separate age groups	Age, sex, ICV	Cortical volume (FreeSurfer), fALFF	15mm	The significance of this relationship was assessed within the FreeSurferframework (mri_glmfit), using cluster-based inference to account for multiple comparisons. To verify the reliability of the findings, several cluster-forming thresholds were tested, ranging from $p < .05$ to $p < .001$ (all tests were two-sided).	No significant relationships were observed between memory improvement and surface-level/vertex-wise cortical volume or cortical fALFF.<br>Similarly, no relationships were found at the MNI voxel-level when investigating noncortical fALFF.

Supplementary 1: UKB data collection details

A. UKB participants' recruitment

The UKB participants were unselected volunteers from the United Kingdom [55]. Exclusion criteria were limited to the presence of metal implant or any recent surgery and health conditions problematic for MRI imaging (e.g. hearing, breathing problems or extreme claustrophobia) [13].

B. T1 weighted and T2 FLAIR image collection

The T1 weighted (T1w) images were acquired over 4:54 minutes, voxel size 1.0x1.0x1.0mm, matrix of 208x256x256mm, using a 3D MPRAGE sequence, sagittal orientation of slice acquisition, R=2 (in plane acceleration factor), TI/TR=880/2000ms [13]. The T2 FLAIR acquisition lasted 5:52 minutes, voxel size 1.05x1.0x1.0 mm, matrix of 192x256x256 voxels, 3D SPACE sequence, sagittal orientation, R=2, partial Fourier 7/8, fat saturated, TI/TR=1800/5000ms, elliptical [13].

C. Participants inclusion and exclusion criteria

We considered the first 10,103 participants of the UK Biobank (UKB) imaging wave. None of the participants withdrew consent after the data were collected. We excluded 231 participants due to T1 images labelled unusable by the UKB or because the FreeSurfer processing failed or did not complete within 48 hours.

D. Sample QC

We excluded pairs of individuals with most similar/dissimilar brain as defined by +-5SD from the mean of the brain-relatedness matrix off-diagonal values. These outlying pairs of participants may bias the LMM estimates and cause convergence problems as REML estimation relies on the brain-relatedness matrix, which corresponds to the variance-covariance matrix of the random effects. We used this stringent cut-off although there may be a more optimal QC threshold that could maximise the sample size in future studies.

We have previously observed in the Human Connectome Project sample that this QC strategy led to the exclusion of a handful of individuals whose processing was flagged using the ENIGMA visual QC protocol [12].

In the UKB, our QC step led to exclude 80.6% of the participants processed using T1w only (vs. 9.9% of the individuals processed using T1w+T2w, p-value<10^{-16}), which confirmed our QC could identify individuals or groups of individuals with outlying brain measurements [17]. In addition, QCed out participants had lower cortical thickness and more extreme ICV, cortical thickness, surface area and subcortical volumes (positive associations with the quadratic terms; p<1e-^{16}). Finally, our QC excluded slightly more males than females (14.6% vs. 10.4%, p-value=4.6e^{-10}) and marginally older participants (63.0 vs. 62.5 years of age, p-value=0.018).

[55] C. Sudlow, J. Gallacher, N. Allen *et al.*, "UK biobank: an open access resource for identifying the causes of a wide range of complex diseases of middle and old age," *PLoS Med,* vol. 12, no. 3, pp. e1001779, Mar, 2015.

[13] K. L. Miller, F. Alfaro-Almagro, N. K. Bangerter *et al.*, "Multimodal population brain imaging in the UK Biobank prospective epidemiological study," *Nat Neurosci,* vol. 19, no. 11, pp. 1523-1536, Nov, 2016.

[12] B. Couvy-Duchesne, L. T. Strike, F. Zhang *et al.*, "A unified framework for association and prediction from vertex-wise grey-matter structure," *Human Brain Mapping,* vol. n/a, no. n/a, 2020.

[17] H. Lindroth, V. A. Nair, C. Stanfield *et al.*, "Examining the identification of age-related atrophy between T1 and T1 + T2-FLAIR cortical thickness measurements," *Sci Rep,* vol. 9, no. 1, pp. 11288, Aug 2, 2019.

Supplementary 2: Details of Statistical Power calculation

We defined statistical power as the True Positive Rate, for a fixed risk alpha (cluster FWER <0.20). In order to achieve a similar control of false positive rate across the different models, we considered a wide range of significance thresholds (down to p<1e-52), all more stringent than the Bonferroni correction. For each significance threshold, we quantified the cluster FWER and TPR. For each model, we reported the largest TPR that satisfied the constraint of cluster FWER < 0.2. We provide below several plots that illustrate the results obtained, and allow the reader to appreciate power for different levels of cluster FWER.

First, we confirmed that lowering the significance threshold (below the Bonferroni one used throughout the analyses) led to a reduction in cluster FWER (**Appendix 2 Figure 1**). Similarly, lowering the significance threshold led to fewer true positive reaching significance, hence a lower TPR (**Appendix 2 Figure 1**).

Finally, we plotted the statistical power for different levels of cluster FWER. The results presented in the main text (cluster FWER<0.2) may be visualised using the vertical dashed line. Overall, the LMM models yielded a greater statistical power than the GLM models.

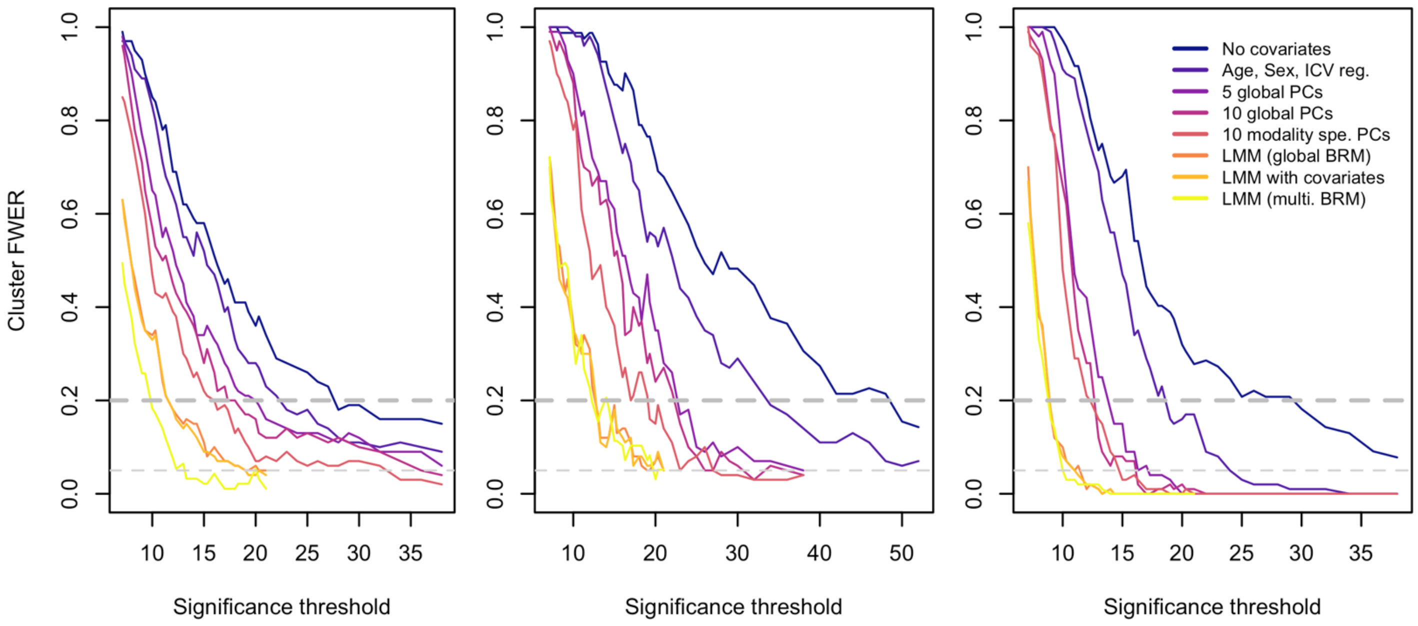

Appendix 2 Figure 1: Evolution of the cluster FWER when lowering the significance threshold.
The x axis is in the logarithmic scale (-log(pvalue significance threshold)). The top dashed line corresponds to a cluster FWER<0.2. The bottom dashed line corresponds to the more traditional alpha level of cluster FWER<0.05. The three panels correspond to the different simulation scenarios (10, 100 and 1000 associated vertices).

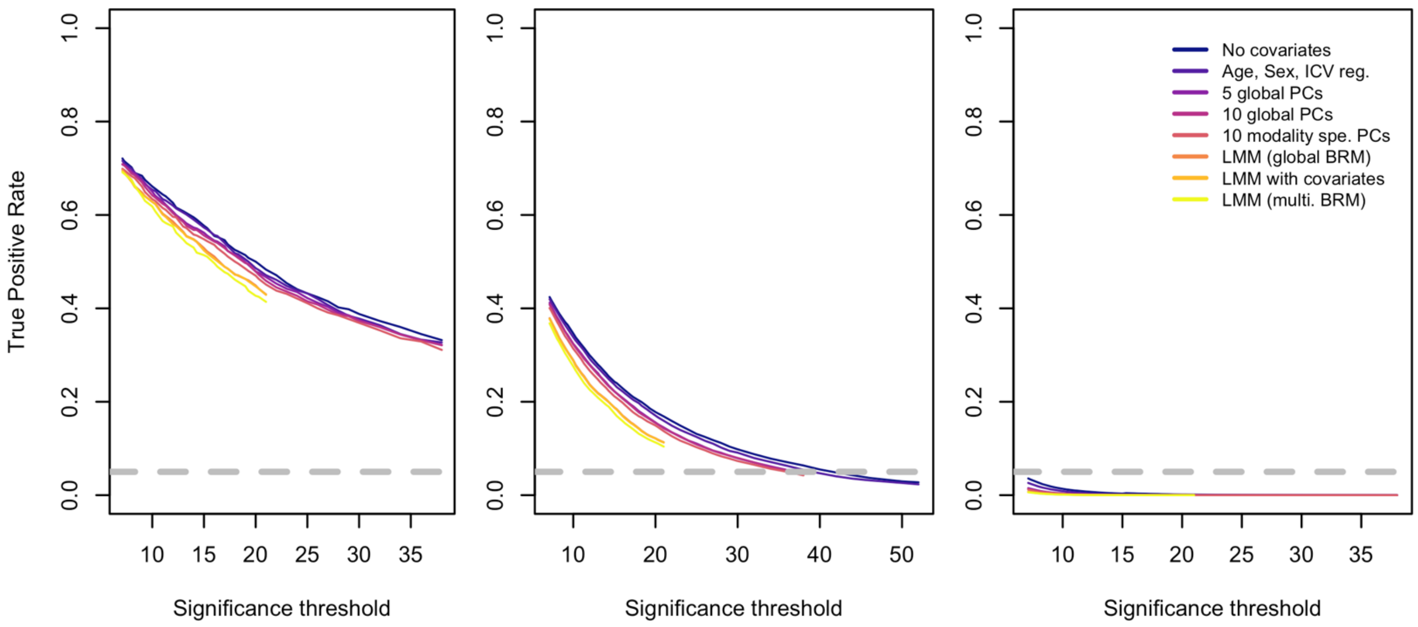

Appendix 2 Figure 2: **Evolution of the True Positive Rate (TPR) when lowering the significance threshold.**
The x axis is in the logarithmic scale (-log(pvalue significance threshold)). The three panels correspond to the different simulation scenarios (10, 100 and 1000 associated vertices).

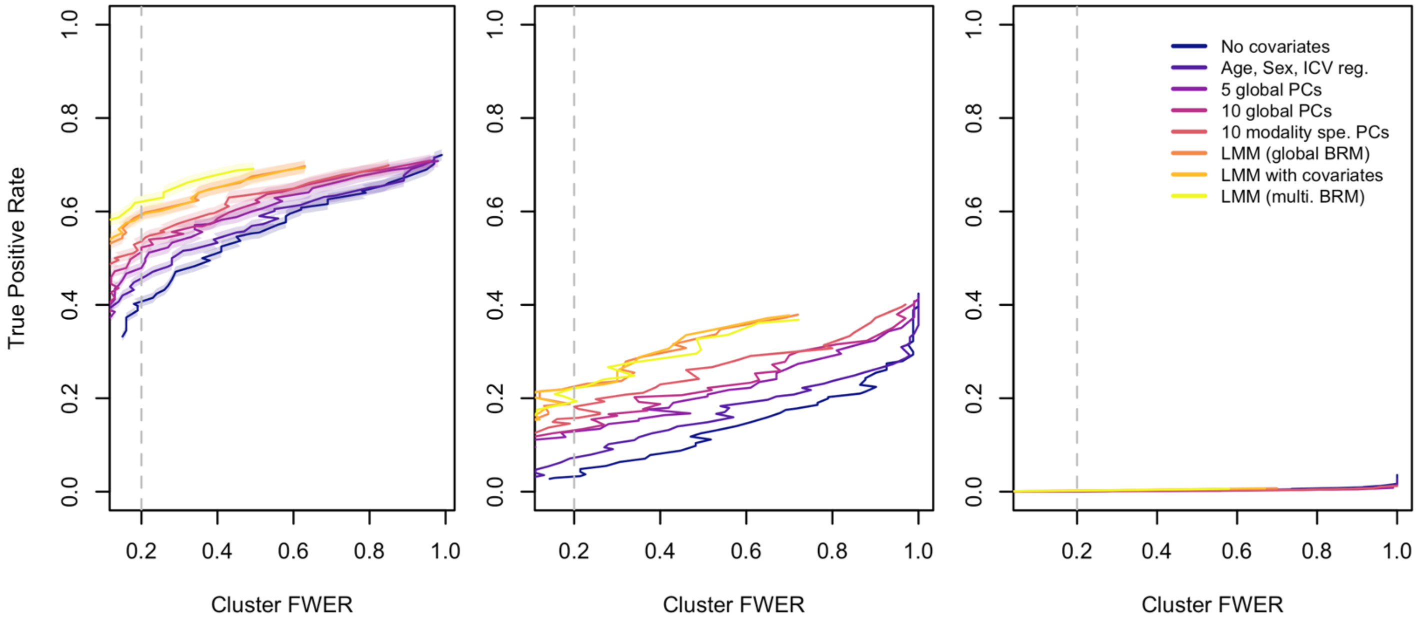

Appendix 2 Figure 3: **Statistical power (True positive rate) for different levels of risk alpha (cluster FWER).**
The vertical dashed line corresponds to cluster FWER<0.2, which corresponds to the results presented in the main text. The three panels correspond to the different simulation scenarios (10, 100 and 1000 associated vertices).
